# Identification of Membrane-expressed CAPRIN-1 as a Novel and Universal Cancer Target, and Generation of a Therapeutic Anti-CAPRIN-1 Antibody TRK-950

**DOI:** 10.1158/2767-9764.CRC-22-0310

**Published:** 2023-04-18

**Authors:** Fumiyoshi Okano, Takanori Saito, Yoshitaka Minamida, Shinichi Kobayashi, Takayoshi Ido, Yasushi Miyauchi, Ukei Wasai, Daisuke Akazawa, Masahiko Kume, Masaki Ishibashi, Ke Jiang, Alexandra Aicher, Christopher Heeschen, Tetsu Yonehara

**Affiliations:** 1 Toray Industries, Inc., New Frontiers Research Laboratories, Kamakura, Kanagawa, Japan.; 2 Kamakura Techno‒Science, Inc., Kamakura, Kanagawa, Japan.; 3 Pancreatic Cancer Heterogeneity, Candiolo Cancer Institute – FPO – IRCCS, Candiolo, Torino, Italy.; 4 Center for Single-Cell Omics and Key Laboratory of Oncogenes and Related Genes, Shanghai Jiao Tong University School of Medicine, Shanghai, P.R. China.; 5 Graduate Institute for Biomedical Sciences Precision Immunotherapy Group China Medical University, North District Taichung City, Taiwan.

## Abstract

Specific targets for cancer treatment are highly desirable, but still remain to be discovered. While previous reports suggested that CAPRIN-1 localizes in the cytoplasm, here we now show that part of this molecule is strongly expressed on the cell membrane surface in most solid cancers, but not normal tissues. Notably, the membrane expression of CAPRIN-1 extended to the subset of highly tumorigenic cancer stem cells and epithelial–mesenchymal transition (EMT)–induced metastatic cancer cells. In addition, we revealed that cancer cells with particularly high CAPRIN-1 surface expression exhibited enhanced tumorigenicity. We generated a therapeutic humanized anti-CAPRIN-1 antibody (TRK-950), which strongly and specifically binds to various cancer cells and shows antitumor effects via engagement of immune cells. TRK-950 was further developed as a new cancer drug and a series of preclinical studies demonstrates its therapeutic potency in tumor-bearing mouse models and safety in a relevant cynomolgus monkey model. Together, our data demonstrate that CAPRIN-1 is a novel and universal target for cancer therapies. A phase I clinical study of TRK-950 has been completed (NCT02990481) and a phase Ib study (combination with approved drugs) is currently underway (NCT03872947) in the United States and France. In parallel, a phase I study in Japan is in progress as well (NCT05423262).

**Significance:**

Antibody-based cancer therapies have been demonstrated to be effective, but are only approved for a limited number of targets, because the majority of these markers is shared with healthy tissue, which may result in adverse effects. Here, we have successfully identified CAPRIN-1 as a novel truly cancer-specific target, universally expressed on membranes of various cancer cells including cancer stem cells. Clinical studies are underway for the anti-CAPRIN-1 therapeutic antibody TRK-950.

## Introduction

Cancer is one of the leading causes of mortality worldwide, and the incidence is still rising (1). In addition to expanding our ability for early detection, enlarging our armamentarium for defeating cancer remains our top priority to improve patient outcomes and survival. There has been tremendous technical progress in antibody-based treatments such as antibody–drug conjugates (ADC), and technology employing the antigen binding moieties of antibodies such as the use of chimeric antibody receptor-T (CAR-T) cells as an immunotherapy for certain types of cancer. These revolutionary therapies have the potential to be significantly more effective than conventional chemotherapeutic agents (2–4), but their success primarily relies on targeting antigens that are truly specific to cancer cells.

To date, antibody-based cancer therapeutics such as ADCs and CAR-T cells only exist for a few established targets. So far, the FDA has approved a total of 12 ADCs and 4 CAR-T cells most of which target hematologic diseases. Only 4 ADCs have been approved for solid cancer targets and FDA-approved CAR-T immunotherapies directed against nonhematologic cancer are currently nonexistent, mostly because the majority of the targets is shared with healthy tissue, which may result in adverse effects, thus limiting their therapeutic window. As such, there is an urgent need for novel and truly cancer-specific targets expressed in a broad range of cancers to develop cancer immunotherapies that are applicable to large populations of patients with solid cancers and having unmet medical needs.

For this purpose, we performed serologic analyses of expression cDNA libraries by the SEREX method (5) using both serum of tumor-bearing dogs and canine testis tissue, allowing us to clone cancer antigens eliciting high-titer antibody-mediated immune responses. In this process, we identified CAPRIN-1 as a novel candidate target for cancer treatment. A previous report had shown CAPRIN-1 to be a cytoplasmic protein without a transmembrane domain (6). The literature has described several functions of cytoplasmic CAPRIN-1 as follows. CAPRIN-1 is expressed during cell activation from a resting state, during cell division, and is also required for cell growth (6, 7). CAPRIN-1 binds to RNA-binding proteins, forming stress granules in the cells (8–16). However, to the best of our knowledge, no report to date has shown that CAPRIN-1 is expressed on the cell membrane surface. On the basis of our initial data using our newly generated anti-CAPRIN-1 antibodies, we reported the possibility that CAPRIN-1 is specifically expressed on cancer cell membranes and could be a target for antibody cancer treatment (17). After our report above, while several reports demonstrated that intracellular expression of CAPRIN-1 positively correlates with cancer progression and poor prognosis, suggesting that CAPRIN-1 could be a diagnostic biomarker and suitable cancer therapeutic target (18–25), we continued to focus on performing a detailed analysis of CAPRIN-1 expression on cancer cell membranes. As a result, we can now reveal that CAPRIN-1 has a novel and distinct expression pattern. While CAPRIN-1 is not expressed on the cell membrane surface of nontransformed cells, it is strongly and reproducibly expressed on the cell membrane surface of many cancer types. Importantly, CAPRIN-1 is also very highly expressed on the membrane surface of highly tumorigenic cancer stem cells. Consistently, cancer cells with particularly high CAPRIN-1 surface expression exhibited enhanced *in vitro* colony-forming activity and *in vivo* tumorigenicity. Together, these data suggest that CAPRIN-1 could be an attractive and widely applicable therapeutic target for cancer, for example, antibody-based treatment approaches. Indeed, the subsequently developed humanized and clinical-grade anti-CAPRIN-1 antibody TRK-950 efficiently eliminates various cancer cells and showed an excellent safety profile in primates. On the basis of these results, a phase I clinical study for TRK-950 has been completed (NCT02990481) and a phase Ib clinical study on the effects of TRK-950 in combination with approved anticancer treatments is currently ongoing (NCT03872947) in the United States and France. In parallel, a phase I clinical study in Japan is in progress as well (NCT05423262).

## Materials and Methods

### Primary Human Immune Cells

Human peripheral blood mononuclear cells (PBMC) were isolated from peripheral blood of healthy donors by centrifugation with Histopaque-1077 (Sigma-Aldrich, catalog no.10771). Human natural killer (NK) cells were isolated from PBMC using the Human NK Cell Isolation Kit (Miltenyi Biotec, catalog no.130-092-657). The purity of NK cells was confirmed to be ≥95% as assessed by flow cytometry using FITC-labeled human CD45 (BioLegend, catalog no. 304006, RRID:AB_314394) and Allophycocyanin (APC)-labeled human CD56 (BioLegend, catalog no. 318310, RRID:AB_604106) antibodies. Peripheral blood was collected and used at Toray Industries, Inc. in conformity with recognized ethical guidelines including the Declaration of Helsinki. Written informed consent was obtained from each donor. The study protocol was approved by Ethics Review Committee on Research Involving Human Biological Subjects, Toray Industries, Inc. (HC2020-15, HC2021-15).

### Primary Cells from Patients

With Institutional Review Board approval, cellular material was collected in conformity with internationally recognized ethical guidelines, including the Declaration of Helsinki. Primary human cancer cells were collected upon patients’ written informed consent. Primary gastric cancer (GC-125, GC-187) and colorectal cancer cells (CRC-515, CRC-1278) were collected at the Candiolo Cancer Institute [Candiolo (TO), Italy] under the ethical approval PROFILING no. 001-IRCC-00IIS-10. Triple-negative breast cancer cells BB6RC37 were collected by the Manchester Cancer Research Centre Biobank (license number: 30,004) ethically approved as a research tissue bank by the South Manchester Research Ethics Committee (Re:07/H1003/161+5) for the Manchester Breast Centre (University of Manchester, Manchester, United Kingdom) as part of the EurOPDX Consortium, which was supported by the European Union's Horizon 2020 research and innovation program under the grant agreement no. 731105 EDIReX (https://www.pdxfinder.org/source/uom-bc/; ref. 26). Pancreatic ductal adenocarcinoma (PDAC) cells SiC-002, SiC-003, SiC-005, SiC-007, SiC-20, SiC-21, SiC-30, and SiC-31 were processed under the Shanghai Jiao Tong University ethical approval number 20130905. Cancer-associated fibroblasts (M146) were collected at the Klinikum Rechts der Isar (Technical University Munich, Munich, Germany) under the Ethical approval number 5510/12. All patients underwent fully written informed consent in accordance with local research ethics committee guidelines.

### Mice

The BT-474–bearing xenograft models were conducted at Oncodesign. Animal housing and experimental procedures were conducted according to the French and European Regulation and the National Research Council Guide for the Care and Use of Laboratory Animals. The animal facility is authorized by French authorities. Animals were maintained in specific pathogen-free (SPF) health status according to the Federation of European Laboratory Animal Science Associations (FELASA) guidelines. Healthy 6-week-old female NOD-SCID (NOD.CB17-Prkdcscid /NcrCrl, RRID:IMSR_JAX:001303) were purchased from Charles River. Other mouse experiments were conducted at Toray. Animal housing and experimental procedures were conducted according to the Guidelines for Animal Experiments of the Research & Development Division, Toray Industries, Inc. Healthy 6-week-old female NOD-SCID mice and BALB/c (RRID:IMSR_JAX:000651) mice were purchased from Charles River Laboratories and bred in the SPF animal facility in individually ventilated cages.

### Cell Culture

The following human and mouse cell lines were obtained from the ATCC: human breast cancer BT-474 (catalog no. HTB-20, RRID:CVCL_0179), human breast cancer MCF7 (catalog no. HTB-22, RRID:CVCL_0031), human breast cancer T-47D (catalog no. HTB-133, RRID:CVCL_0553), human breast cancer SK-BR-3 (catalog no. HTB-30, RRID:CVCL_0033), human urinary bladder cancer SW780 (catalog no. CRL-2169, RRID:CVCL_1728), human pancreas cancer Panc10.05 (catalog no. CRL-2547, RRID:CVCL_1639), human colon cancer HT-29 (catalog no. HTB-38, RRID:CVCL_0320), human colon cancer HCT116 (catalog no. CCL-247, RRID:CVCL_0291), human colon cancer COLO 205 (catalog no. CCL-222, RRID:CVCL_0218), human stomach cancer NCI-N87 (catalog no. CRL-5822, RRID:CVCL_1603), human prostate cancer 22Rv1 (catalog no. CRL-2505, RRID:CVCL_1045), human lung cancer A549 (catalog no. CCL-185, RRID:CVCL_0023), human lung cancer NCI-H2228 (catalog no. CRL-5935, RRID:CVCL_1543), human liver cancer Hep3B (catalog no. HB-8064, RRID:CVCL_0326), human pharynx cancer FaDu (catalog no. HTB-43, RRID: CVCL_1218), human ovarian cancer TOV-21G (catalog no. CRL-11730, RRID:CVCL_3613), human uterus cancer HEC-1-A (catalog no. HTB-112, RRID:CVCL_0293), human malignant melanoma Malme-3M (catalog no. HTB-64, RRID:CVCL_1438), human malignant melanoma SK-MEL-5 (catalog no. HTB-70, RRID:CVCL_0527), human malignant melanoma A-375 (catalog no. CRL-1619, RRID:CVCL_0132), human leukemia Jurkat (catalog no. TIB-152, RRID:CVCL_0367), human leukemia MV-4-11 (catalog no. CRL-9591, RRID:CVCL_0064), human leukemia NALM6 (catalog no. CRL-3273, RRID:CVCL_UJ05), and human leukemia THP-1 (catalog no. TIB-202, RRID:CVCL_0006), human normal retina ARPE-19 (catalog no. CRL-2302, RRID:CVCL_0145), human normal skin HFF-1 (catalog no. SCRC-1041, RRID:CVCL_3285), human normal lung WI-38 (catalog no. CCL-75, RRID:CVCL_0579), mouse breast cancer 4T1 (catalog no. CRL-2539, RRID:CVCL_0125), and mouse subcutaneous connective tissue L-929 (catalog no. CCL-1, RRID:CVCL_0462).

The following human cell lines were obtained from Japanese Collection of Research Bioresources (JCRB): human cholangiocarcinoma KKU-213 (catalog no. JCRB1557, RRID:CVCL_M261), human breast cancer MRK-nu-1 (catalog no. JCRB0628, RRID:CVCL_1428), human liver cancer HepG2 (catalog no. JCRB1054, RRID:CVCL_0027), human ovarian cancer MCAS (catalog no. JCRB0240, RRID:CVCL_3020), human pancreas cancer MIA PaCa-2 (catalog no. JCRB0070, RRID:CVCL_0428), human lymphoma Daudi (catalog no. JCRB9071, RRID:CVCL_0008), human leukemia KCL-22 (catalog no. JCRB1317, RRID:CVCL_2091), human lymphoma Raji (catalog no. JCRB9012, RRID:CVCL_0511), human lymphoma U937 (catalog no. JCRB9021, RRID:CVCL_0007), and mouse monocyte-macrophage J774.1 (catalog no. JCRB0018, RRID:CVCL_4770) and monkey COS-1 (catalog no. JCRB9082, RRID:CVCL_0223).

The following human cell lines were obtained from European Collection of Authenticated Cell Culture: human uterine cancer Ishikawa (catalog no. 99040201, RRID:CVCL_2529), human normal thyroid Nthy-ori 3-1 (catalog no. 90011609, RRID:CVCL_2659) and mouse myeloma SP2/0 (catalog no. 85072401, RRID:CVCL_2199). 293FT cells (catalog no. R70007, RRID:CVCL_6911) were obtained from Thermo Fisher Scientific. Human ovarian cancer OVCAR3 (catalog no. RCB2135, RRID:CVCL_0465) cells were obtained from RIKEN BRC. All cell lines were cultured according to the cell bank's instructions.

Cells were cryopreserved at early passages (<5) and used from fresh stock vial for each experiment and never continued the culture for more than 2 months. These cells were not further authenticated by our laboratory; however, routine confirmation of *in vitro* growth properties, morphology provided evidence of correct cell identity.

*Mycoplasma* testing was performed using TaKaRa PCR *Mycoplasma* Detection Set (TaKaRa, catalog no. 6601) at the time of stock vial preparation.

### SEREX

The healthy canine testis tissue and the serum of tumor-bearing beagle dogs were obtained from the Veterinary ME Research Center, Japan. Messenger RNA was purified from canine testis tissue using the Oligotex dT30 mRNA Purification Kit (Takara, catalog no. 9086), and a cDNA phage library was generated using the ZAP-cDNA Gigapack III Gold Cloning Kit (Agilent, catalog no. 200450). XL1-Blue MRF’ competent cells (Agilent, catalog no. 200230) were infected with the phages in the cDNA library and seeded on NZY agar plates (Quality Biological, catalog no. 340-124-231). After incubation for 4 hours at 42°C, we placed a nitrocellulose membrane Hybond-C Extra (Cytiva, catalog no. RPN82E) containing 20 mmol/L Isopropyl β D-thiogalactoside (Nacalai Tesque, catalog no.19742-94) on the plate for 4 hours at 37°C to transfer the expressed proteins to the membrane. After the membrane was blocked with 0.5% skim milk in TBS for 1 hour at room temperature, the serum of a malignant mammary tumor of a beagle dog was incubated for 3 hours at room temperature. The membrane was washed four times with TBS-T (TBS with 0.1% Tween 20) and incubated with Goat anti-Dog IgG antibody (BETHYL Laboratories, catalog no. A40-123, RRID:AB_66900) diluted 3,000 times for 1 hour at room temperature. Positive plaques were detected by NBT/BCIP solution (Roche, catalog no. 11-681-451-001) and collected. We isolated phage plasmids from positive plaques using the ZAP-cDNA Gigapack III Gold Cloning Kit and conducted sequence analysis of these plasmids using T3 and T7 primers.

T3 sequence: ATTAACCCTCACTAAAGGGAA
T7 sequence: TAATACGACTCACTATAGGG

### Recombinant Human CAPRIN-1 Protein

Total RNA was purified from cancer cell lines using TRIzol Reagent (Invitrogen, catalog no. 15596026), and cDNA was generated using SuperScript III First-Strand Synthesis System for RT-PCR (Invitrogen, catalog no. 18080-051). This cDNA was used as a template for cloning the full length of human CAPRIN-1. The sequence of cloning primer is shown below.

Forward: ACGCACGGGATCCGCCGCCACCATGCCCTCGGCCACCAGCC
Reverse: CCGCCGCTCGAGTTAATTCACTTGCTGAGTGTTCATTTGCGGC

The PCR product was integrated into the pET-30a (+) vector (Novagen, catalog no. 69909). This plasmid vector was transformed into BL21 (DE3) competent cells (Novagen, catalog no. 69450), which expressed His-tagged CAPRIN-1 protein. Protein was purified with Ni Sepharose 6 Fast Flow (Cytiva, catalog no. 17531801) and HiTrap Q HP (Cytiva, catalog no. 29051325).

### Generation of Anti-CAPRIN-1 Antibodies

#### Rabbit Polyclonal Antibody

The rabbit anti-CAPRIN-1 polyclonal antibody was generated by Biologica Co. Recombinant human CAPRIN-1 protein (0.5 mg) was mixed with same amount of Freund's Incomplete Adjuvant solution and administered to a rabbit subcutaneously. Immunization was performed once every 2 weeks for four times in total. After the end of immunization, whole blood was collected to obtain serum, and anti-CAPRIN-1 polyclonal antibody (pAb-1) was purified using rProtein A Sepharose Fast Flow (Cytiva, catalog no. 17127902). The control antibody was purified from peripheral blood of rabbits to which the antigen was not administered.

#### Rabbit Monoclonal Antibody (mAb)

The generation of rabbit anti-CAPRIN-1 mAb was conducted at Epitomics, Inc. Recombinant human CAPRIN-1 protein (1 mg) was mixed with Freund's Complete Adjuvant and administered to a rabbit subcutaneously. Immunization was performed once every 2 weeks for 10 times in total. The increase of antibodies raised against CAPRIN-1 protein in the serum was measured by ELISA. After a sufficient increase in titer, we performed a final immunization, dissected the spleen, and fused splenocytes with rabbit myeloma cells to generate rabbit hybridoma cells. Screening of the rabbit hybridoma supernatants was performed by two methods, namely reactivity to CAPRIN-1 protein by ELISA and binding to the surface of cancer cell membrane by flow cytometry. MAbs (mAb-1, mAb-3, mAb-5, mAb-6, mAb-7, mAb-8, and mAb-9) were purified and obtained using rProtein A Sepharose Fast Flow from cultured supernatant.

#### Mouse mAb

We generated mouse anti-CAPRIN-1 mAbs by the following procedure. Recombinant human CAPRIN-1 protein (0.1 mg) was mixed with the same amount of TiterMax Gold Adjuvant (Sigma-Aldrich, catalog no. T2684) and administered to the peritoneal cavity of BALB/c mouse. Immunization was performed once a week for four times in total. After a confirmed increase in titer by ELISA, the spleen was dissected and fused with the mouse myeloma cell line SP2/0 to generate mouse hybridoma cells. Screening was performed by two methods, namely reactivity to CAPRIN-1 protein by ELISA and binding to the surface of cancer cell membrane by flow cytometry. mAb (mAb-2) was purified and obtained using rProtein A Sepharose Fast Flow from the supernatant of hybridoma, which we cultured for 1 week in Hybridoma-SFM medium (Gibco, catalog no. 12045-076).

#### Chicken/Human Chimera Antibody

The generation of chicken anti-CAPRIN-1 mAb and chicken/human chimera antibody were conducted at Hiroshima Bio-Medical Co., Ltd. Recombinant human CAPRIN-1 protein (0.1 mg) was mixed with Freund's Complete Adjuvant and administered to the peritoneal cavity of chicken. Immunization was performed once every 3 weeks and three times in total. After a confirmed increase in titer by ELISA, we performed the final immunization, extracted the spleen, and fused the splenocytes with chicken B cells to generate chicken hybridoma cells. Screening of reactivity to CAPRIN-1 was conducted as indicated above. mAbs were purified and obtained from the supernatants of the cultured hybridoma cells. On the basis of the chicken antibody, chicken/human chimera antibody (mAb-4) was generated by genetic recombination.

#### Humanized Antibody (TRK-950)

The generation of humanized anti-CAPRIN-1 antibody was conducted at Epitomics, Inc. Total mRNA was extracted from the selected rabbit anti-CAPRIN-1 mAb (mAb-1) hybridoma to generate cDNA via reverse transcription reaction. The variable regions of heavy chain and light chain were cloned from cDNA by PCR. Parts of the frame region in the variable region were replaced with human IgG1 sequence. This sequence was fused with the Fc portion of human IgG1a to generate an expression plasmid (27). This plasmid was transfected into HEK293 cells, and humanized CAPRIN-1 antibody (TRK-950) was purified from the supernatant using HiTrap Protein G HP (Cytiva, catalog no. 17040403).

### Flow Cytometry

Cells were detached from culture flasks using 2.5 mmol/L Ethylene Diamine Tetraacetic Acid (EDTA) (DOJINDO, catalog no. 345-01865), washed with PBS, and resuspended in FACS buffer (PBS with 1% FBS) for use. The detached cells (1 × 10^5^ cells) were mixed with first antibody to a final concentration of 1 to 10 μg/mL and incubated for 1 hour at 4°C. 1st antibody; anti-CAPRIN-1 antibodies, that is, mAb-1 to -9, mAb-10 (Invitrogen, catalog no. 703658, RRID:AB_2784596), pAb-2 (Sigma-Aldrich, catalog no. HPA018126, RRID:AB_1849929) and negative control antibodies, that is, rabbit IgG (FUJIFILM, catalog no. 148-09551, RRID:AB_2920644), mouse IgG2b (MBL, catalog no. M076-3M2, RRID:AB_1953018), human IgG (Sigma-Aldrich, catalog no. I4506, RRID:AB_1163606). After washing with FACS buffer, we added the second antibody, that is, FITC Goat anti-rabbit IgG (BD Biosciences, catalog no. 554020, RRID:AB_395212), Alexa Fluor 488 anti-rabbit IgG(H+L) (Invitrogen, catalog no. A21206, RRID:AB_2535792), Alexa Fluor 647 anti-rabbit IgG (BioLegend, catalog no. 406414, RRID:AB_2563202), Alexa Fluor 488 F(ab')2 anti-mouse IgG(H+L) (Invitrogen, catalog no. A11017, RRID:AB_143160), and Alexa Fluor 488 anti-human IgG(H+L) (Invitrogen, catalog no. A11013, RRID:AB_141360), respectively. The second antibody was adjusted to a final concentration of 10 μg/mL and incubated for 1 hour at 4°C in a dark place. Following washes with FACS buffer, we added propidium iodide solution (DOJINDO, catalog no. 341-07881) at a final concentration of 0.1 μg/mL or Fixable Viability Stain 450 (BD Biosciences, catalog no. 562247, RRID:AB_2869405) for dead/live cell discrimination. Samples were run on a flow cytometer (FACSCalibur, FACSVerse, and Fortessa, BD Biosciences), followed by analysis using FlowJo (BD Biosciences).

### ELISA

We coated 20 ng/well of recombinant CAPRIN-1 protein to Nunc MaxiSorp flat-bottom 96-well plate (Thermo Fisher Scientific, catalog no. 44-2404-21) for overnight binding at 4°C, followed by blocking with 3% skim milk in PBS for 1 hour at room temperature. The first antibody was incubated for 1 hour at room temperature. Each isotype IgGs were used as a negative control. We then washed the plate with PBS-T and added the second antibody, that is, horseradish peroxidase (HRP) anti-rabbit IgG (BioLegend, catalog no. 406401, RRID:AB_2099368), HRP anti-mouse IgG (Abcam, catalog no. 6789, RRID:AB_955439), and HRP anti-human IgG (Abcam, catalog no. ab6858, RRID:AB_955433), and incubated for 1 hour at room temperature. TMB substrate (Nacalai tesque, catalog no. 05299-54) was used for color development, and stopped the reaction with 1N H_2_SO_4_, prior to measuring optical density at 450–595 nm using a Plate Reader (Multiskan FC, Thermo Fisher Scientific).

### Western Blot Analysis

The harvested cells were washed with PBS, suspended in RIPA Buffer (Nacalai tesque, catalog no. 16488-34) containing Protease Inhibitor Cocktail (Nacalai tesque, catalog no. 25955-24), incubated on ice for 15 minutes, and centrifuged (12,000 × *g*, 10 minutes, 4°C). The supernatant was collected as a cell lysate. Next, we added Sample Buffer Solution (Nacalai tesque, catalog no. 09499-14) containing 100 mmol/L Dithiothreitol (DTT) prior to boiling, and ran the samples on 4%–20% gradient gels (TEFCO, catalog no. 01-022-10) for SDS-PAGE, and performed transfer on Immobilon-P PVDF membranes (Millipore, catalog no. IPVH00010). Blocking with 5% skim milk in TBS-T for 1 hour at room temperature. The membranes were incubated with 0.5 μg/mL anti-CAPRIN-1 antibodies, 0.3 μg/mL anti-GAPDH antibody (Abcam, catalog no. ab9485, RRID:AB_307275), 0.1 μg/mL anti-Sodium Potassium ATPase antibody (Abcam, catalog no. ab76020, RRID:AB_1310695) in 5% skim milk/TBS-T for 1 hour at room temperature, after washing the membrane four times with TBS-T, incubated with the second antibody, that is, HRP anti-rabbit IgG, HRP anti-mouse IgG, and HRP anti-human IgG for 1 hour at room temperature. Signals were detected using Western Lightning Plus-ECL (PerkinElmer, catalog no. NEL104001EA) on a FUSION SOLO.6S.EDGE (Vilber Lourmat) chemiluminescence imaging system.

### Plasma Membrane Isolation

Plasma membranes were purified from BT-474 cells using the Minute Plasma Membrane Protein Isolation Kit (Invent, catalog no. SM-005). Isolation was performed according to the manufacturer's instructions.

### Competitive Inhibition Assay

The anti-CAPRIN-1 antibodies were incubated at 10 μg/mL with 250 μg/mL of rCAPRIN-1 protein or BSA for 30 minutes at room temperature. Then, we added 1 × 10^5^ cells to them and incubation of 1 hour at 4°C. The samples were washed with FACS buffer, the second antibody was added and analyzed by Flow Cytometry.

### CAPRIN-1 Knockdown

Stealth RNAi siRNA for CAPRIN-1 (Invitrogen, catalog no. HSS106231, HSS106233) or Stealth RNAi siRNA Negative Control (Invitrogen, catalog no. 12935300) were transfected to cancer cells using Lipofectamine RNAiMAX Transfection Reagent (Invitrogen, catalog no. 13778150). Transfection was performed according to the manufacturer's instructions.

### CAPRIN-1 Stably Expressing Cells

The generation of vector pRP (Exp)-EGFP/Puro-CMV (RRID:Addgene_41841) which coded the full length of human CAPRIN-1 and control vector was conducted by Vector Builder. These vectors were transfected to 293FT cell using Lipofectamine 3000 (Invitrogen, catalog no. 3000001). The transfected cells were added 3 μg/mL of Puromycin (Gibco, catalog no. A11138-03) for selection. After 4 weeks of culture, the expression of CAPRIN-1 was evaluated by Western blot analysis and flow cytometry. We used parent vector transfected cells as mock.

### Tissue and Cell Immunostaining

We used formalin-fixed paraffin-embedded cancer and normal tissues on human tissue microarrays (US Biomax, Inc., Shanghai Outdo Biotech Co., Ltd., BioChain Inc., and Diagnostic Oncology CRO, LLC). For cell immunostaining, we fixed cells with formalin, agar, or paraffin embedded them, prior to prepare 2-μm-thick sections. The sections were deparaffinized with xylene, rehydrated with ethanol, and pressure cooked with 0.01 mol/L citrate buffer (pH 6.0) to retrieve the antigen. Intrinsic peroxidase activity was blocked by 3% H_2_O_2_. To avoid nonspecific staining, samples were treated with 20% normal goat serum/PBS for 20 minutes prior to be incubated with 2 μg/mL mAb-1 antibody overnight at 4°C. We used the MACH4 Universal HRP-Polymer Detection System (BIOCARE MEDICAL, catalog no. BRR4012H) for detection. The proportion of the cells that positively stained for CAPRIN-1 in the cytosol and cell membrane was categorized as follows: no reactivity (0), faint/barely perceptible reactivity (1+), weak to moderate reactivity (2+), and strong reactivity (3+).

### Sphere Culture

The cells were resuspended at a density of 1 × 10^4^ cells/mL in DMEM/F-12 supplemented with 20 ng/mL FGF (PeproTech, catalog no. 100-18C), 0.4% amphotericin B, 1% penicillin/streptomycin, 2% B-27 supplement (Invitrogen, catalog no. 12587010), and 200 mmol/L of l-glutamine. Spheres were harvested after 7 days using a 40 μm cell strainer and then dissociated using TrypLE Express (Gibco, catalog no. 12605-028).

### Mammosphere Assay

Single-cell suspension was prepared by manual disaggregation (25 gauge needle) and a total of 500 cells/cm^2^ were plated in appropriate poly (2-hydroxyethylmethacrylate)–coated tissue culture plates in mammosphere medium [phenol red-free DMEM/F12 (Gibco, catalog no. 21041)] containing B-27 supplement, 20 ng/mL rEGF (Sigma-Aldrich, catalog no. E9644) and penicillin/streptomycin. Cells were cultured for 7 days before mammospheres with diameter greater than 40 μm were obtained, and dissociated using TrypLE Express.

### Detection of Cancer Stem Cells

Cells were resuspended in FACS buffer and incubated with mAb-1 or TRK-950. For mAb-1, cells were first incubated with mAb-1 at 4°C for 45 minutes, followed by a washing step with FACS buffer and incubated with Alexa Fluor 647 goat anti-Rabbit IgG (Invitrogen, catalog no. A-21244, RRID:AB_2535812) or Alexa Fluor 488 anti-Rabbit IgG secondary antibody for 45 minutes at 4°C. For TRK-950, cells were first incubated with TRK-950 for 30 minutes at 4°C, then washed once with FACS buffer and incubated with Alexa Fluor 647 goat anti-human IgG secondary antibody (Invitrogen, catalog no. A-21445, RRID:AB_2535862) for 45 minutes at 4°C. When CD133 was used as a surface marker for the detection of cancer stem cells, above cells were blocked with Flebogamma (5 μL per 1 million cells) for 10 minutes at 4°C, and then incubated with anti-human CD133 PE antibody (Miltenyi Biotec, catalog no. 130-112-352, RRID:AB_2654909) for 30 minutes at 4°C. After a final washing step, DAPI was added, and cells were immediately analyzed on the Attune NxT flow cytometer (Thermo Fisher Scientific) or the LSR Fortessa flow cytometer (BD Biosciences).

For detection of autofluorescence, cells were resuspended in 10 mL of RPMI complete media including 30 μmol/L of Riboflavin (Sigma-Aldrich, catalog no. R7649), incubated for 48 hours, and then stained for CAPRIN-1 using TRK-950 as outlined above. The autofluorescence signal was detected using a 488 nm blue laser for excitement equipped with the filters 530/40 and 580/30 for detection on the Attune NxT flow cytometer.

### Preparation of Macrophage Conditioned Medium

Monocyte-derived human macrophage cultures were established from PBMCs collected from healthy donors. PBMCs were cultured in Iscove's modified Dulbecco's medium (Gibco, catalog no. 12440061) supplemented with 10% human AB serum (Sigma-Aldrich, catalog no. H4522). To generate “M2-like tumor -associated” macrophages, adherent PBMC-derived monocytes were polarized the day after initial seeding with 0.5 ng/mL of MCSF (PeproTech, catalog no. 300-25) for 48 hours. To generate macrophage conditioned media (MCM), macrophages were washed with PBS and DMEM/F12 supplemented with 2% B-27, 2 mmol/L l-Glutamine, 50 U/mL penicillin/streptomycin, and 20 ng/mL FGF was added for 48 hours. Media were then collected, centrifuged, and the supernatant used for induction of EMT.

### Induction of EMT and Immunofluorescent Staining

SiC-003 cells in 100 μL RPMI1640 were seeded in 12-well chamber slides (Ibidi, catalog no. 81201) and cultured overnight. Medium was replaced with 200 μL MCM for induction of EMT or the same volume of standard RMPI1640 as control and cultured for 48 hours (28). Then, cells were washed in PBS, and immediately fixed in 4% paraformaldehyde for 10 minutes at room temperature. Cells were again washed with PBS three times for 10 minutes at room temperature and permeabilized with 0.1% Triton X-100 in PBS for 10 minutes at room temperature.

After cells were washed with PBS once for 10 minutes at room temperature, slides were incubated with Blocking Solution (PBS with 5% BSA) for 1 hour at room temperature, followed by Alexa Fluor 750 anti-E-Cadherin antibody (Novus Biologicals, catalog no. NBP2-89336AF750) and Alexa Fluor 594 anti-Vimentin antibody (Abcam, catalog no. ab154207) 1:100 in Blocking Solution overnight at 4°C in a humidified chamber. Then, cells were washed with PBS three times for 5 minutes at room temperature and nuclei stained with 1 μg/mL DAPI in PBS for 15 minutes at room temperature. Images were recorded using the Operetta platform (PerkinElmer).

### Cell Sorting

HEC-1-A, SK-MEL-5, and Malme-3M cells were sorted into two populations with high and low expression levels of CAPRIN-1 using mAb-1 by flow cytometer (FACS Aria Fusion, BD Biosciences). Sorted cells were washed twice with culture media and cultured at 37°C.

### Soft Agar Colony Formation Assay

Anchorage-independent growth of sorted cells was evaluated by soft agar colony formation assay. The sorted cells were passaged within 2 weeks before processing. The cells (1 × 10^4^ cells) were incubated in a top layer of 0.4% noble agar (Difco, catalog no. 214220) in DMEM with 10% FBS. The suspension was overlaid on a bottom layer of 0.6% noble agar in DMEM with 10% FBS in a 6-well plate. DMEM was added to each well and cultured at 37°C for 2 to 4 weeks. Each well was scanned automatically using the BZ-X810 system (Keyence), and the whole well was reconstructed from the scanned images using the BZ-X Analysis software (Keyence). Colonies larger than 20,000 μm^2^ were counted using the Hybrid Cell Count software (Keyence). The experiments were conducted three times independently.

### Cell Proliferation Assay

Proliferation of sorted cells was analyzed using CellTiter-Glo Luminescent Cell Viability Assay (Promega, catalog no. G7570) according to the manufacturer's instructions. The luminescence was detected using SpectraMac iD3 (Molecular Devices).

The proliferation of siRNA-transfected cells and TRK-950–treated cells were evaluated using 3H-thymidine (PerkinElmer, catalog no. NET027Z). The detached cells were seeded at 5 × 10^3^ cells/well in 96-well plates and incubated at 37°C with CO_2_. After 3 and 6 days, we added 37 Bq of 3H-thymidine per well. After 18 hours, cells were harvested using a FilterMate Cell Harvester (Perkin Elmer) and Unifilter GF/B (Perkin Elmer, catalog no. 6005177), and 3H-thymidine was detected by TopCount NXT (Perkin Elmer).

### Binding Affinity

The binding affinity analysis of TRK-950 against CAPRIN-1 protein using Biacore 3000 (Cytiva) was conducted at LSI Medience. Recombinant CAPRIN-1 protein was adjusted to 5 μg/mL using 10 mmol/L sodium acetate pH 4.5, and solid-phased on Sensor Chip CM5 using surface density 417 response units. BSA (397 RU) was used as reference. The antibody as the analyte was adjusted to 2.5, 1.25, 0.625, 0.313, and 0.156 μg/mL using HSB-EP (0.01 mol/L HEPES pH 7.4, 0.15 mol/L NaCl, 3 mmol/L EDTA, 0.005% Surfactant P20) and sent to the surface of the sensor chip under the following conditions: flow rate, 20 μL/minute; binding time, 120 seconds; dissociation time, 120 seconds; and temperature, 25°C. Kinetic data were analyzed using BIAevaluation ver4.01. For each binding curve, the response obtained using reference surface was subtracted. The antibody binding fitted a 1:1 Langmuir binding model using global fitting.

### Antibody-dependent Cellular Cytotoxicity Activity

The antibody-dependent cellular cytotoxicity (ADCC) activity of TRK-950 was evaluated by Chromium-51 release assays to detect cytotoxicity mediated through human and mouse NK cells. Mouse NK cells were isolated from spleens of NOD-SCID mice using the Mouse NK Cell Isolation Kit (Miltenyi Biotec, catalog no. 130-096-892). Target cells were suspended in medium and incubated with 100 μL of 37 MBq/mL Chromium-51 (PerkinElmer, catalog no. NEZ030S) for 1 hour at 37°C CO_2_ incubator. After washing, 2 × 10^3^ cells/well target cells were seeded to 96-well V-Bottom plate, and antibody, effector cells were added. After 5 hours incubation at 37°C CO_2_ incubator, the plate was centrifuged (300 × *g*, 5 minutes), and the supernatant transferred to LumaPlate-96 (PerkinElmer, catalog no. 6006633), and dried. The Cr-51 radiation dose (cpm) of the LumaPlate-96 was measured by TopCount NXT. For a spontaneous release, target cells alone, and for a maximum release, completely dead target cells by adding 2% Triton. Cytotoxicity (%) was calculated using the following formula based on the Cr-51 dose released from the target cells:



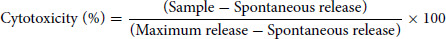



The ADCC activity of TRK-950 via human and monkey PBMCs was conducted at Translational Drug Development (TD2). We used human PBMCs (AllCells, catalog no. PB003F) and Cynomolgus Monkey PBMCs (Zen-Bio) as effector cells.

### Antibody-dependent Cellular Phagocytosis Activity

We evaluated the antibody-dependent cellular phagocytosis (ADCP) activity of TRK-950 by phagocytosis of cancer cells through cancer cell lines, human monocyte-derived macrophages, mouse bone marrow (BM)-derived macrophages, tumor-infiltrating macrophage.

To obtain human macrophages were isolated from human PBMC and cultured in 50 ng/mL human GMCSF (PeproTech, catalog no. 300-03) at 37°C in a CO_2_ incubator. Medium was replaced 2 days later, and the cells were harvested after a total of 5 more days. The induction of human macrophages was confirmed by flow cytometry using PE anti-human CD11b antibody (BioLegend, catalog no. 101208, RRID:AB_312791) and PE anti-human CD14 antibody (BioLegend, catalog no. 301806, RRID:AB_314188).

To obtain mouse BM macrophages, BM cells were collected from femora of NOD-SCID mice and cultured for 7 days in RPMI1640 medium with 10% FBS and 1% PS to which 30% conditioned medium from L-929 cells was added. The induction of mouse macrophages was confirmed by flow cytometry using APC/Cyanine7 anti-mouse/human CD11b (BioLegend, catalog no. 101226, RRID:AB_830642) and APC anti-mouse F4/80 antibodies (BioLegend, catalog no. 123116, RRID:AB_893481).

To obtain mouse tumor-infiltrating macrophages, tumor masses from BT-474–bearing NOD-SCID mice were digested using Tumor Dissociation Kit (Miltenyi Biotec, catalog no. 130-096-730) and cancer cells were removed by density gradient separation with Histopaque-1077 and macrophages were isolated by MACS with anti-F4/80 MicroBeads UltraPure (Miltenyi Biotec, catalog no. 130-110-443). The purity was checked by flow cytometry using APC/Cyanine7 anti-mouse/human CD11b and APC anti-mouse F4/80 antibodies.

Human neutrophils were isolated from healthy donors using the MACSxpress Whole Blood Neutrophil Isolation Kit (Miltenyi Biotec, catalog no. 130-104-434), and monkey neutrophils were isolated using the CD66abce MicroBead Kit (Miltenyi Biotec, catalog no. 130-092-393) from cynomolgus monkey at Envigo.

The target cancer cells were incubated at a final concentration of 20 ng/mL of Calcein-AM (DOJINDO, catalog no. C396) at 37°C with CO_2_ for 30 minutes, and washed with medium prior to use.

The target cells, effector cells, and antibodies were added to Ultra-Low Attachment 96-well plates (Costar, catalog no. CLS7007) and incubated for 4 hours at 37°C with CO_2_. After the incubation, the plate was centrifuged (300 × *g*, 5 minutes) and washed to collect cells from the wells and analyzed by flow cytometry. Phagocytosis (%) was calculated using the following formulas based on the ratio of effector cells:



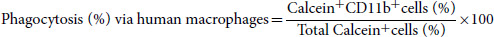





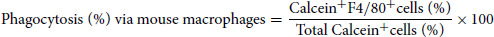





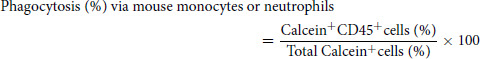





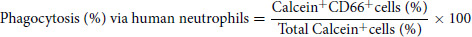





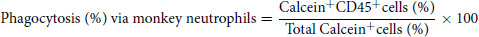



### Xenograft Model

The tumorigenicity of high and low CAPRIN-1–expressing cells was evaluated in xenograft models. The sorted cells were allowed to grow for 4 days before injection. HEC-1-A (2.5 × 10^5^ cells), SK-MEL-5 (1 × 10^5^ cells), and Malme-3M (1 × 10^5^ cells) were resuspended with 100 μL of DMEM containing 25% Matrigel Basement Membrane Matrix (Corning, catalog no. 354234), and injected subcutaneously into the right or the left flanks of the same NOD-SCID mice under isoflurane anesthesia. We used 10 mice for each sorted cell group. We defined the day of the cancer cell injection as day 0. The tumor diameter was measured once a week using calipers. The tumor volume was calculated using the following formula:



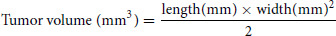



The antitumor activity of TRK-950 was evaluated using NOD-SCID mice bearing BT474 cells and COLO 205 cells. BT-474 (2 × 10^7^ cells) and COLO 205 (5 × 10^6^ cells) were suspended in 200 μL of DMEM containing 50% Matrigel Basement Membrane Matrix (Corning, catalog no. 356237) and injected subcutaneously into the right flank of NOD-SCID mice under isoflurane anesthesia. We defined the day of the cancer cell injection as day 0. The mice were randomized into each group by mean tumor volume when tumors reached a mean volume of 100 to 200 mm^3^ and treatment started. The COLO 205–bearing mice were treated from day 0. The administration substances; vehicle (PBS pH 7.3–7.4), TRK-950 in PBS. The administration routes of substances were intravenous injection into the caudal vein. Tumor volume data up to humanitarian euthanasia were adopted.

### *In Vivo* Imaging

Mouse 4T1 cells (2 × 10^6^ cells) were suspended in 100 μL PBS and subcutaneously injected to the back of BALB/c mice under isoflurane anesthesia. IRDye800-conjugated TRK-950 was generated using the IRDye800CW Protein Labeling Kit (LI-COR, catalog no. 928-38040) according to the manufacturer's instructions. The drug-to-antibody ratio of the generated IRDye800-conjugated TRK-950 was 1.45. After 10 days inoculation, the IR800Dye-labeled TRK-950 (1 mg/kg) was administered via tail vein injection and evaluated the distribution and accumulation in the tumor. As control, no tumor-bearing BALB/c mice were used. *In vivo* IRDye800 fluorescence distribution was measured by Pearl Trilogy (LI-COR) 1, 3, and 6 days after TRK-950 administration.

### Cell Fixation and Permeabilization

The cells were harvested using 2.5 mmol/L EDTA, washed with PBS, fixed with 4% formaldehyde for 20 minutes at room temperature, and permeabilized with 0.1% TritonX-100 in PBS (v/v) for 15 minutes at room temperature.

### Toxicity Study

The toxicity study of TRK-950 was conducted at Envigo in accordance with the applicable sections of the United Kingdom Animals (Scientific Procedures) Act 1986 m Amendment Regulations 2012. TRK-950 was administered to cynomolgus monkeys by intravenous slow bolus injection at dose levels of 3, 25, or 200 mg/kg on days 1, 8, and 15 (3 males and females, respectively). Recoverability was monitored for an 8-week recovery period. Animals were evaluated for clinical observations, body weights, food consumption, body temperatures, ophthalmic examinations, hematology, clinical chemistry, urinalysis, organ weights, gross and microscopic pathology (days 22 and 72). Blood samples were collected throughout the study for toxicokinetics, anti-drug antibody (ADA), and cytokine release analysis on days 1, 8, 15, 22, 29, 43, and 71. Safety pharmacology parameters were incorporated and included the assessment of respiratory function (respiratory rate), cardiovascular function (electrocardiograms, blood pressure, pulse rates), and neurobehavioral screening to evaluate potential effects on the central nervous system. In addition, cytokine release (IFNγ, IL1β, IL6, and TNFα) and immunophenotyping were included.

### Quantification and Statistical Analysis

In tumor growth studies for HEC-1-A, SK-MEL-5, Malme-3M, and COLO 205 cells bearing models; Mann–Whitney *U* tests, for BT-474–bearing models; Kruskal–Wallis tests, and in other figures unpaired two-tailed Student *t* tests were conducted using GraphPad Prism Ver 9.0 (GraphPad Software) to calculate the *P* value, *, *P* < 0.05; **, *P* < 0.01; ***, *P* < 0.001.

### Data Availability Statement

The data generated in this study are available upon reasonable request from the corresponding author.

## Results

### CAPRIN-1 is Expressed on the Surface of the Cancer Cell Membrane

**FIGURE 1:**
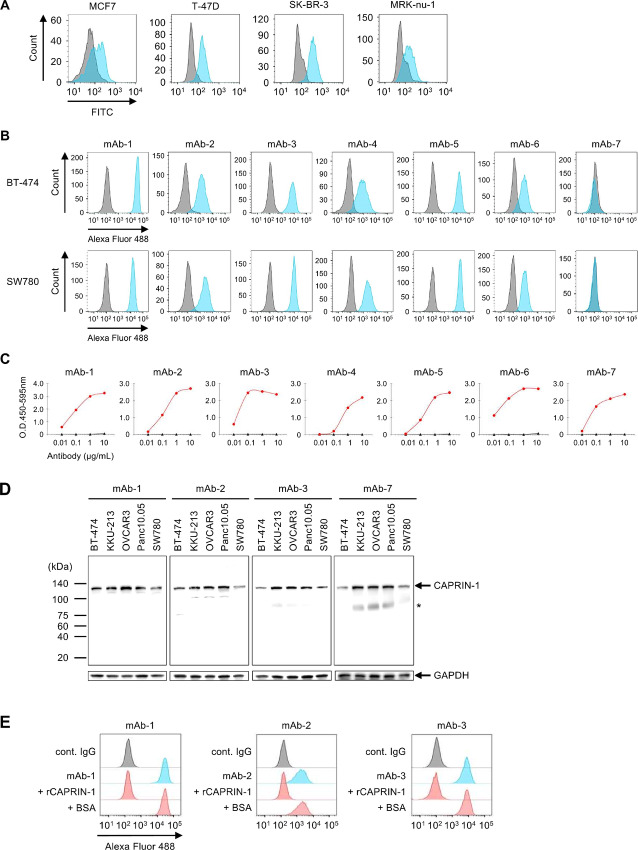
Anti-CAPRIN-1 antibodies binding to the membrane surface of cancer cells. **A,** Anti-CAPRIN-1 polyclonal antibody pAb-1 binds to the surface of cancer cells. Flow cytometry histograms for CAPRIN-1 polyclonal antibody (blue) versus control rabbit IgG (gray). FITC anti-rabbit IgG was used as secondary antibody. **B,** Binding activity of anti-CAPRIN-1 mAbs to the surface of cancer cells. Flow cytometry histograms for anti-CAPRIN-1 mAbs mAb-1 to mAb-7, and control IgG (gray). Alexa Fluor 488 anti-rabbit IgG, anti-mouse IgG, or anti-human IgG were used as secondary antibodies. **C,** Anti-CAPRIN-1 antibodies bind to recombinant CAPRIN-1 protein in ELISAs. Red line: anti-CAPRIN-1 antibodies, black line: control IgG. **D,** Expression of CAPRIN-1 protein in cell lysates from cancer cells was detected by Western blot analysis using the anti-CAPRIN-1 antibodies. GAPDH was used as loading control. *, nonspecific band. **E,** Competition assay with rCAPRIN-1 protein. Binding of mAb-1, 2, 3 to the cell surface of BT474 cells was analyzed by flow cytometry. Flow cytometry histograms for mAb-1, 2, 3 (blue), control IgG (gray), mAb-1, 2, 3 competed with rCAPRIN-1 or BSA (red). BSA was used as a negative control.

A result of the SEREX screening using both serum of mammary gland tumor-bearing dog and canine testis tissue, we identified CAPRIN-1 as a novel candidate target for cancer treatment. We generated a rabbit anti-CAPRIN-1 polyclonal antibody (pAb-1) and tested its binding to the surface membrane of four human breast cancer cell lines, using flow cytometry. Although CAPRIN-1 is reportedly an intracellular protein, antibody pAb-1 unexpectedly binds to the surface of all tested cancer cells (Fig. 1A), suggesting that CAPRIN-1 may be expressed on the surface of breast cancer cells. To further explore whether CAPRIN-1 is indeed expressed on cancer cell membranes, we next generated a large variety of mAbs binding to CAPRIN-1 protein from rabbits, mice, and chicken. Most of the generated antibodies, such as mAb-7, mAb-8, and mAb-9, bound to recombinant CAPRIN-1 protein but did not bind to the surface of cancer cell membranes (Fig. 1B and C; Supplementary Fig. S1A and S1B). Moreover, commercially available anti-CAPRIN-1 antibodies, such as mAb-10 and pAb-2, also did not bind to the surface of cancer cell membranes. However, other antibodies, such as the anti-CAPRIN-1 antibodies mAb-1 to 6, did indeed bind to both CAPRIN-1 protein and the membrane surface of cancer cells such as BT-474 (breast cancer) and SW780 (bladder cancer; Fig. 1B and C).

To further confirm whether antibodies that bound to the surface of cancer cell membranes specifically recognize CAPRIN-1, we conducted Western blot analysis with cell lysates and a single band was detected at approximately 120 kDa (Fig. 1D). Moreover, we investigated the specificity of antibody binding by performing competition assays. Recombinant CAPRIN-1 protein inhibited the binding of anti-CAPRIN-1 antibodies to the surface of cancer cell membranes (Fig. 1E; Supplementary Fig. S1C). These results strongly indicated that the generated antibodies specifically recognize CAPRIN-1 expressed on the surface of cancer cell membranes.

**FIGURE 2:**
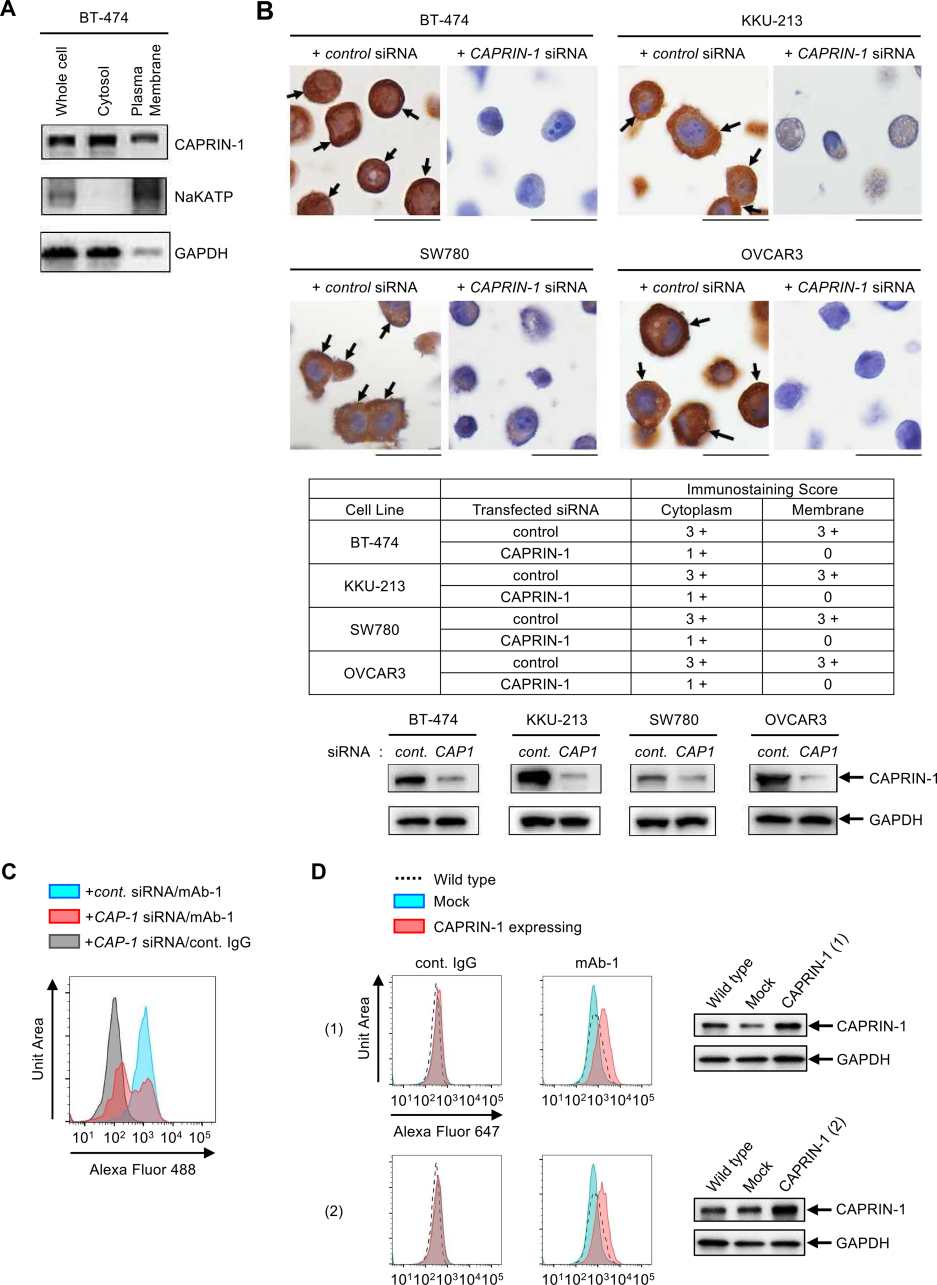
CAPRIN-1 is expressed on the membrane surface of cancer cells. **A,** Detection of CAPRIN-1 in the plasma membrane versus cytoplasmic fraction of BT-474 cells by Western blot analysis using mAb-1. NaKATP, plasma membrane marker; GAPDH, cytoplasmic marker. **B,** Top: Expression of CAPRIN-1 in cancer cells transfected with *CAPRIN-1* or *control* siRNA was visualized by IHC using mAb-1. Arrows indicate representative stained membranes. Scale bar: 20 μm. Middle: Immunostaining scores of CAPRIN-1 on cancer cell membrane and cytosol are provided in bottom. Score mean expression level using mAb-1, 0: negative, 1+: slight, 2+: moderate, 3+: marked. Lower: CAPRIN-1 knockdown was confirmed by Western blot analysis using mAb-1. **C,** CAPRIN-1 expression on the surface of OVCAR3 cells following CAPRIN-1 knockdown was assessed by flow cytometry. Blue: *control* siRNA transfected cells stained with mAb-1; red: *CAPRIN-1* siRNA transfected cells stained with mAb-1; gray: *CAPRIN-1* siRNA transfected cells stained with control IgG. **D,** Detection of CAPRIN-1 in 293FT cells stably expressing CAPRIN-1. Left: Flow cytometry for CAPRIN-1 stably expressing cells (red), mock (blue), or wild-type cells (black dotted line) stained with mAb-1 or control IgG. Alexa Fluor 647 anti-rabbit IgG was used as secondary antibody. Right: CAPRIN-1 expression in respective cells was analyzed by Western blot analysis using mAb-1. CAPRIN-1 (1) and (2): CAPRIN-1 stably expressing cells clone.

To further confirm that CAPRIN-1 is indeed expressed on cancer cell membranes, we separated BT-474 cells into cytosolic and membrane fractions for evaluation by Western blotting. The results indicated that CAPRIN-1 was detected both in cytosol and cell membranes (Fig. 2A). Both mAb-1, which binds to the surface of cancer cell membranes, as well as mAb-7, which does not bind to the membrane surface, showed equal binding patterns once the cells were fixed and permeabilized (Supplementary Fig. S1D). Furthermore, mAb-1 immunostaining of BT-474, SW780, KKU-213 (cholangiocarcinoma), and OVCAR3 (ovarian cancer) cells revealed intense and consistent staining of cytoplasm and cell membranes. Upon knockdown of *CAPRIN-1*, the staining of each component of the cell was strongly diminished or even abrogated (Fig. 2B). Consistently, *CAPRIN-1* knockdown reduced the binding of mAb-1 to OVCAR3 cells as evaluated via flow cytometry (Fig. 2C). As a result of the knockdown of CAPRIN-1 in cancer cells, cell growth was inhibited, which is consistent with previous reports (Supplementary Fig. S2A; refs. 6, 7), in addition, dead cells increased in some cell lines (Supplementary Fig. S2B). In contrast, when we introduced the CAPRIN-1 expression vector in 293FT cells to generate cell clones stably expressing CAPRIN-1, we observed increased binding of mAb-1 to the membrane surface (Fig. 2D). These results indicated that CAPRIN-1 is expressed not only in the cytoplasm but also on cell membranes of cancer cells and that some of our antibodies, for example, mAb-1, are capable of specifically detecting both cytoplasmic and membrane CAPRIN-1.

### CAPRIN-1 is Expressed on Various Types of Cancer Tissues but not on Normal Tissues

**FIGURE 3:**
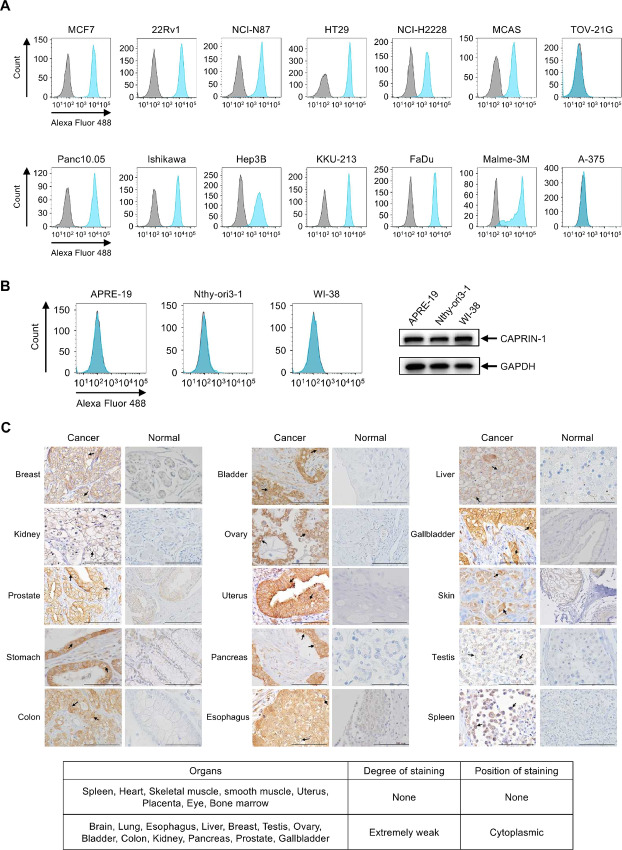
CAPRIN-1 is exclusively expressed on cell membrane surfaces of various types of cancer tissues. CAPRIN-1 expression on the surface of solid cancer cell membranes (**A**) and normal cell membranes (**B**) were analyzed by flow cytometry using mAb-1 (blue) and control IgG (gray). Alexa Fluor 488 anti-rabbit IgG was used as secondary antibody. The following human cancer cells were used: breast cancer MCF7, prostate cancer 22Rv1, stomach cancer NCI-N87, colon cancer HT29, lung cancer NCI-H2228, ovarian cancer MCAS and TOV-21G, pancreas cancer Panc10.05, uterine cancer Ishikawa, liver cancer Hep3B, cholangiocarcinoma KKU-213, pharynx cancer FaDu, melanoma Malme-3M, and A-375. CAPRIN-1 expression in normal cells was analyzed by Western blot analysis using mAb-1 (B-right). **C,** CAPRIN-1 expression of cancer versus normal tissues on tissue microarrays were analyzed by IHC using mAb-1. Arrows indicate representative membranes stained with mAb-1. Scale bar: 100 μm. Bottom: Expression status of CAPRIN-1 on various normal tissues.

We analyzed CAPRIN-1 expression on various types of cancer cells using mAb-1 for flow cytometry. We found that CAPRIN-1 was expressed on the membrane surface of most cancer cell lines and some cell lines, such as TOV-21G (ovarian cancer) and A-375 (melanoma), were negative, although there were not so many negative cancer cell lines (Fig. 3A). Conversely, we did not find the cell surface expression of CAPRIN-1 on normal cells ARPE-19, Nthy-ori 3-1 and WI-38 (nontransformed cell lines derived from normal tissues). And we did not detect CAPRIN-1 surface expression in a comprehensive set of established hematologic cancer cell lines, although we detected CAPRIN-1 in the cell lysates of both normal and hematologic cancer cells by Western blot analysis (Fig. 3B; Supplementary Fig. S3). Furthermore, the expression of CAPRIN-1 in cancer tissue and normal tissue arrays was analyzed via IHC using mAb-1. As shown in Fig. 3C (representative IHC images of tissues), CAPRIN-1 was expressed on the membrane of various cancer tissues but hardly expressed on the cell membranes of normal tissues. Weak cytoplasmic CAPRIN-1 expression could be detected by mAb-1 in several normal tissues (Fig. 3C, bottom). Positive ratio of CAPRIN-1 on cell membrane was remarkably high in various cancer tissues (Table 1).

**TABLE 1:** Positive ratio of CAPRIN-1 on cell membrane in various cancer tissues

Tumor organ	Breast	Kidney	Prostate	Stomach	Colon	Bladder
Positive counts/total (% positive)	255/323 (79%)	145/170 (85%)	154/170 (91%)	99/150 (66%)	146/227 (64%)	66/88 (75%)
Tumor organ	Ovary	Uterus	Pancreas	Esophagus	Liver	Gallbladder
Positive counts/total (% positive)	50/81 (62%)	34/55 (62%)	47/53 (89%)	25/49 (51%)	26/36 (72%)	20/24 (83%)
Tumor organ	Melanoma	Testis	Spleen	Lung	Bile duct	
Positive counts/total (% positive)	20/25 (80%)	7/8 (88%)	5/8 (63%)	126/187 (67%)	23/30 (77%)	

NOTE: The number of tissues which CAPRIN-1 was expressed on cancer cell membrane and total number of tested and positive percentage in IHC using mAb-1.

### CAPRIN-1 is Strongly Expressed on Primary Human Cancer (Stem) Cells

**FIGURE 4:**
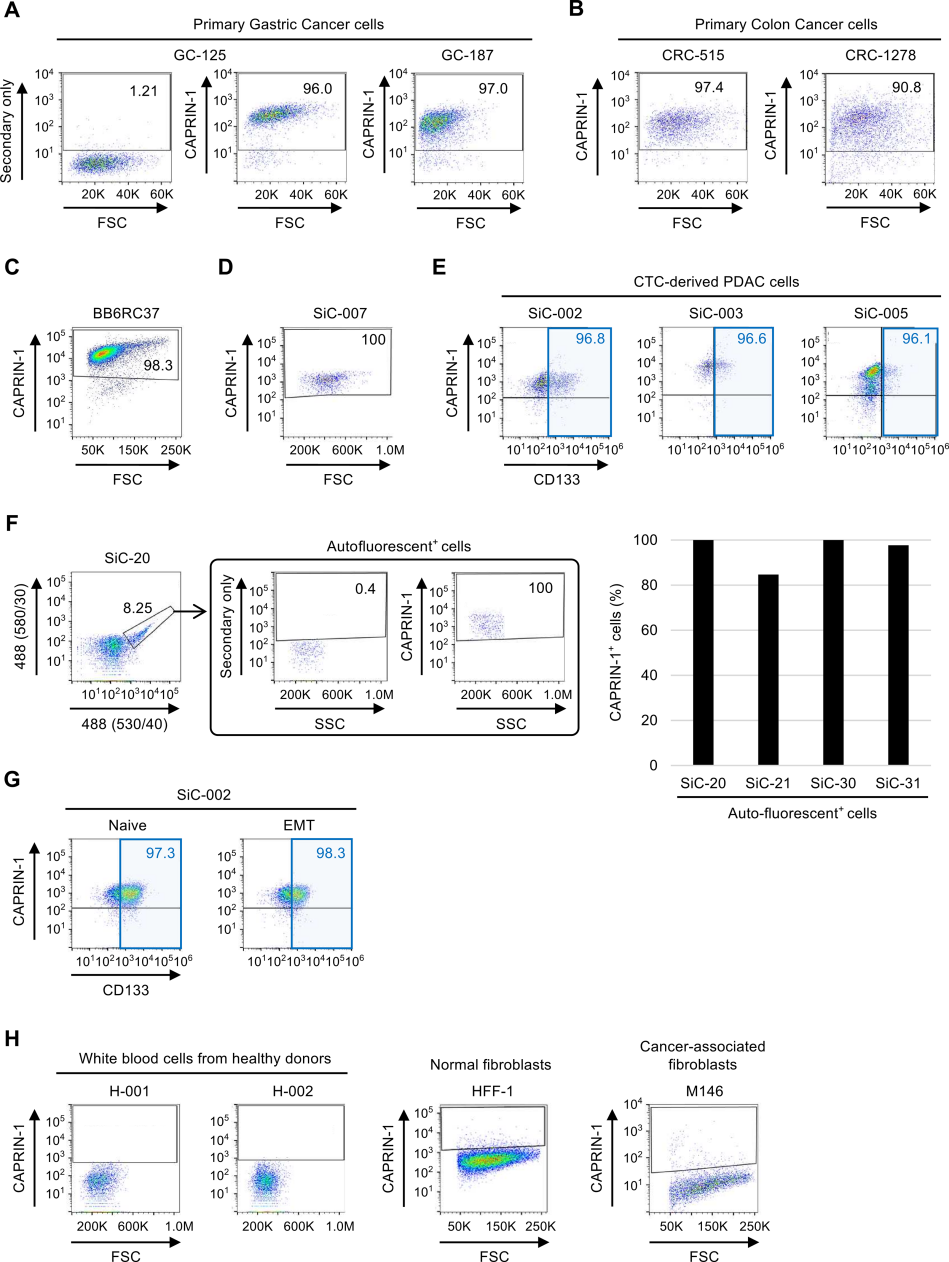
CAPRIN-1 is strongly expressed on the membrane surface of primary human cancer (stem) cells. Flow cytometry analysis of cell surface expression of CAPRIN-1 using mAb-1 (**A–C**) and TRK-950 (**D–G**). **A,** CAPRIN-1 expression of primary gastric cancer cells (GC-125 and GC-187) derived from an individual patient with gastric cancer. **B,** CAPRIN-1 expression of primary colon cancer cells (CRC-515 and CRC-1278) derived from individual patients. **C,** CAPRIN-1 expression of primary triple-negative breast cancer cells in mammosphere culture conditions (BB6RC37) obtained from an individual patient with breast cancer. **D,** CAPRIN-1 expression of primary pancreatic cancer cells in sphere culture conditions (SiC-007) derived from an individual patient with pancreatic cancer. **E,** CAPRIN-1 and CD133 expression of pancreatic cancer cells (SiC-002, 003, 005) derived from individual patients with metastatic pancreatic cancer. **F,** Gating strategy for the subpopulation of autofluorescence^+^ cancer stem cells from advanced pancreatic cancer patients (left). Percentage of CAPRIN-1^+^ cells among autofluorescence^+^ cells derived from individual patients with metastatic pancreatic cancer (right). **G,** CAPRIN-1 expression of pancreatic cancer cells (SiC-002) before and after induction of EMT following treatment with MCM for 48 hours. **H,** CAPRIN-1 expression of white blood cells derived from healthy human donors (H-001, H-002), normal human foreskin fibroblast (HFF-1), and human pancreatic cancer-associated fibroblasts (M146). The percentage of CAPRIN-1^+^ cells relative to all single live cells is depicted as insert (A–D). The percentage of CAPRIN-1^+^ CD133^+^ cells relative to all single live CD133^+^ cells is depicted as insert (E and G).

We next evaluated CAPRIN-1 surface expression via flow cytometry on various primary human cancer cells. The analysis of cells derived from patients with gastric and colorectal cancer demonstrated that CAPRIN-1 was strongly expressed on virtually all cell membrane surfaces (Fig. 4A and B).

Expression of CAPRIN-1 could also be detected on cancer cells derived from a patient with breast cancer and PDAC cells cultured in sphere conditions (Fig. 4C and D), suggesting that CAPRIN-1 is also expressed in cells enriched for highly tumorigenic cancer stem cells (29, 30). Therefore, we next investigated the expression of CAPRIN-1 on the contained subset of cancer stem cells identified by the surface marker protein CD133 (29), which revealed strong and consistent expression of CAPRIN-1 on these particularly aggressive and treatment-resistant cells (Fig. 4E). These data were further corroborated by a detailed investigation of CAPRIN-1 expression on cancer stem cells identified by their inherent feature of autofluorescence (31). Analysis of the auto-fluorescent subset of cancer stem cells in four different primary cultures derived from patients with advanced-stage PDAC revealed that CAPRIN-1 was expressed on the membrane surface of virtually all cells, with the exception of one patient for which we found 84.7% of cancer stem cells to be positive for CAPRIN-1 (Fig. 4F).

To demonstrate that CAPRIN-1 is not downregulated during EMT (28), we analyzed its surface expression in PDAC cells before and after the induction of EMT using MCM (32). As expected, E-Cadherin was downregulated and Vimentin was upregulated in PDAC cells that had undergone EMT in response to exposure to MCM (Supplementary Fig. S4). Notably, our data show that the strong CAPRIN-1 surface expression remained unchanged, irrespective of the state of the cells related to EMT and stemness (Fig. 4G).

Importantly, CAPRIN-1 could not be detected on the membrane surface of white blood cells isolated from healthy volunteers, normal human fibroblasts, and cancer-associated fibroblasts from patients with PDAC (Fig. 4H). These results indicated that CAPRIN-1 is strongly and consistently expressed on primary human cancer cells, including the subset of cancer stem cells. Lack of expression on nontransformed cells suggests that CAPRIN-1 represents a promising target for cancer immunotherapies with a large therapeutic window due to low risk for adverse reactions related to non-cancer targeting.

### Correlation of CAPRIN-1 Expression Levels on the Cell Surface with Tumor Cell Growth *In Vitro* and *In Vivo*

**FIGURE 5:**
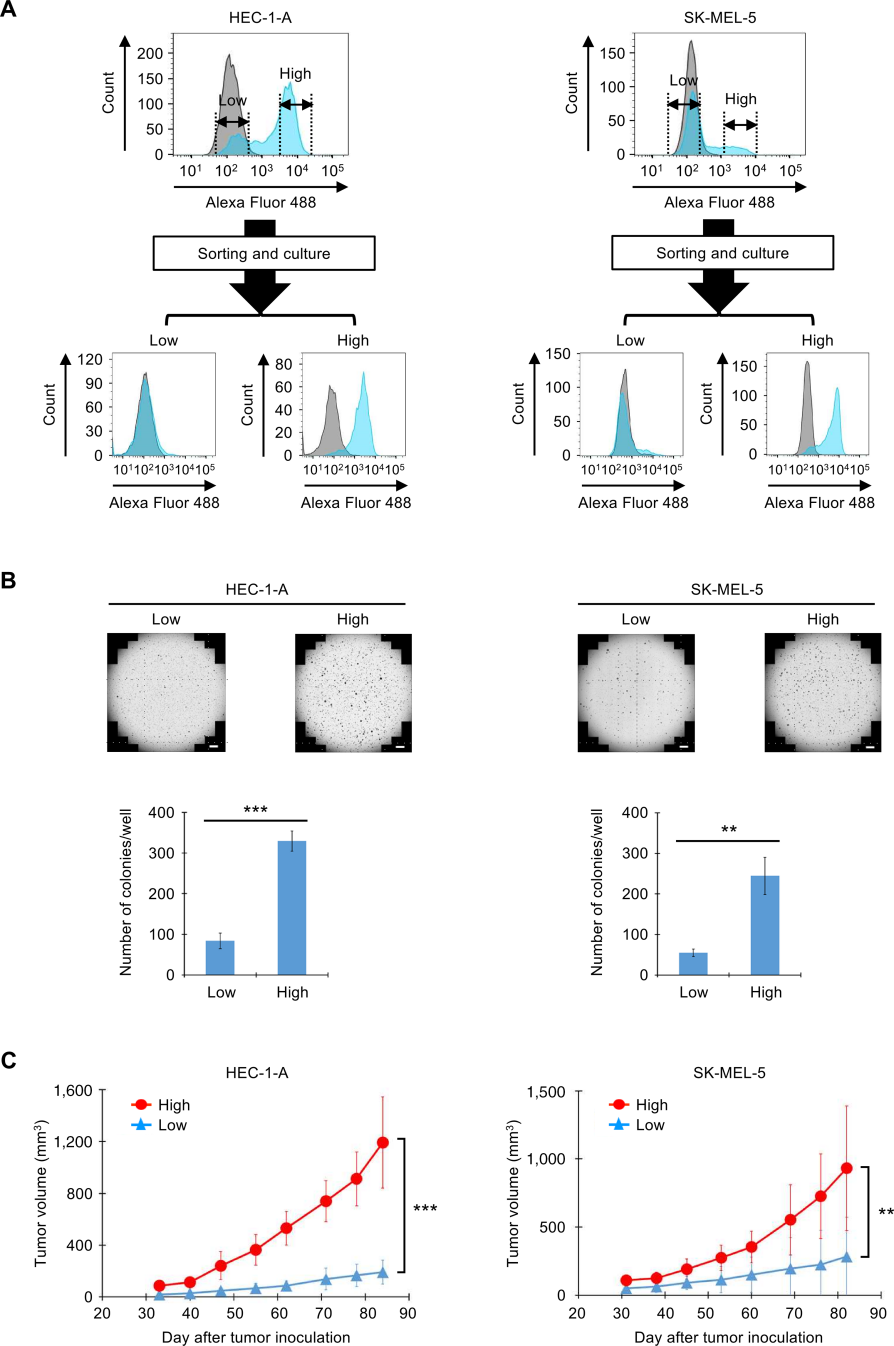
Correlation of CAPRIN-1 expression levels on the cell surface with tumor cell growth *in vitro* and *in vivo*. **A,** HEC-1-A and SK-MEL-5 cells were sorted to CAPRIN-1^high^ and CAPRIN-1^low^ cells and were analyzed by flow cytometry using mAb-1 (blue) or control IgG (gray). Top: Histograms of unsorted cells. Bottom: Histograms of CAPRIN-1^high^ and CAPRIN-1^low^ cells which were cultured for 10–13 days after sorted. **B,** Top: Micrograph of soft agar colony formation assay using the sorted cells. Scale bar, 2 mm. Bottom: The number of colonies larger than 20,000 μm^2^. Data are provided as mean ± SD; *n* = 3. The experiments were conducted three times independently and the same results were obtained. **C,** Tumor growth curves of NOD-SCID mice bearing CAPRIN-1^high^ (red) and CAPRIN-1^low^ (blue) cells. Tumor measurement started about 30 days after injection. Data are given as mean ± SD; *n* = 10/group. Significance calculated using two-tailed Student *t* test (B), Mann–Whitney *U* tests (C); **, *P* < 0.01; ***, *P* < 0.001.

As aforementioned, we evaluated the expression of CAPRIN-1 on the membrane surface in numerous cancer cells and found that some cells such as HEC-1-A (uterine), SK-MEL-5 (melanoma), and Malme-3M (melanoma) contain subpopulations with varying expression levels for CAPRIN-1 (Fig. 5A). To study the potential functional implications of this heterogeneity, cells were sorted into two populations with high and low expression levels of CAPRIN-1. After culturing the cells again for at least 1 week after sorting, the expression levels of CAPRIN-1 remained unchanged (Fig. 5A; Supplementary Fig. S5A), suggesting a stable phenotype. The expression level was analyzed using several anti-CAPRIN-1 antibodies. Indeed, CAPRIN-1^high^ (high CAPRIN-1 membrane expression) cells showed enhanced anchorage-independent growth in soft agar colony-forming assay (Fig. 5B), whereas no obvious difference was observed for their growth rate in regular adherent conditions (Supplementary Fig. S5C). When identical numbers of CAPRIN-1^high^ and CAPRIN-1^low^ (low CAPRIN-1 membrane expression) cells were implanted into NOD-SCID (NOD.CB17-Prkdcscid/NcrCrl) mice, CAPRIN-1^high^ cells resulted in much faster tumor growth (Fig. 5C; Supplementary Fig. S5B). These results indicated that the cells with a high expression of CAPRIN-1 on the membrane surface bear higher *in vivo* tumorigenicity. To rule out contamination in the cancer cells used, multiple genes known to be expressed in each cancer cell were selected from information in the database and expression was evaluated. As a result, expression profiling of the original unsorted cells versus the cells sorted for CAPRIN-1 levels showed consistent expression levels for various genes, thereby reassuring that the cell populations were derived from the same parental clones (Supplementary Fig. S5D).

Next, to investigate whether the expression levels of CAPRIN-1 on the membrane surface of cells are affected by a hypoxic state, which is one of the general features of the intratumoral environment (33), the aforementioned cell lines with high and low expression fractions of CAPRIN-1 on the membrane surface of cells were cultured under hypoxic conditions. The results showed that the proportion of CAPRIN-1^high^ cells increased and the proportion of CAPRIN-1^low^ cells decreased in hypoxic cultures compared with normal cultures (Supplementary Fig. S5E). Of note, SK-MEL-5 caused cell death under hypoxic conditions and could not be used for the evaluation.

### CAPRIN-1 Expression on the Cell Membrane Surface Increases Due to Stressful Stimuli to Cancer Cells

Knowing that cytoplasmic CAPRIN-1 migrates and accumulates in stress granules in response to stressful stimuli (12), we exposed cancer cells to the Endoplasmic Reticulum (ER) stress inducer Tunicamycin and evaluated whether the expression level of CAPRIN-1 on the cancer cell membranes changes. Indeed, we found that Tunicamycin increased surface CAPRIN-1 expression in cancer cells with virtually no CAPRIN-1 expression at baseline. The stress-induced surface expression of CAPRIN-1 was abrogated following CAPRIN-1 knockdown, confirming that the increased expression was due to CAPRIN-1 (Supplementary Fig. S6).

### Generation of Humanized Anti-CAPRIN-1 Antibody TRK-950

**FIGURE 6:**
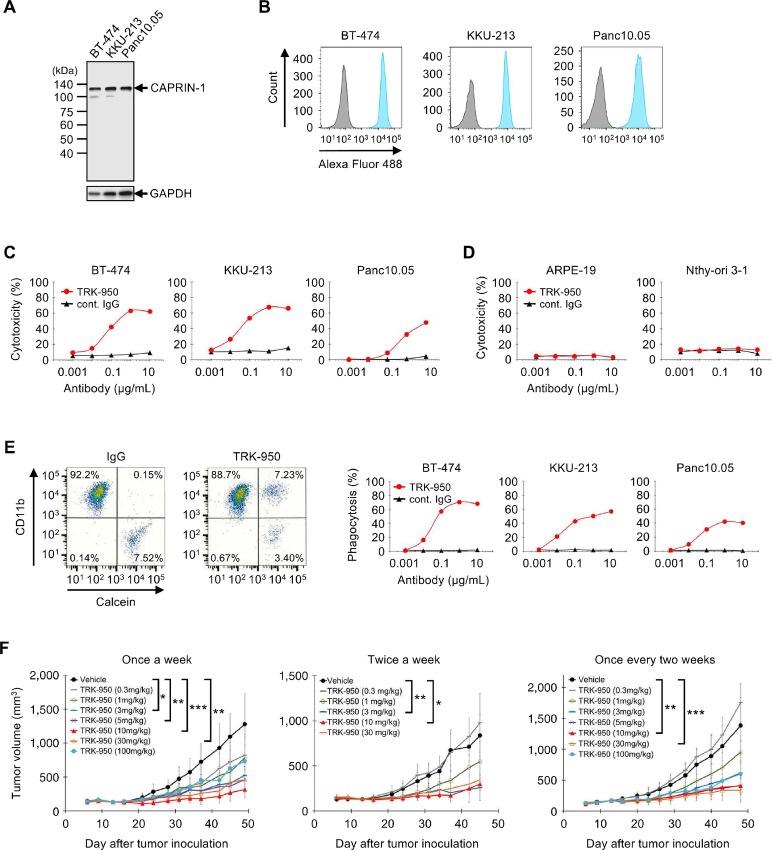
Characterization of the humanized anti-CAPRIN-1 antibody TRK-950. **A,** CAPRIN-1 in cell lysates of cancer cells was detected by Western blot analysis using TRK-950. **B,** Flow cytometry analysis of CAPRIN-1 expression on cell membrane of cancer cells using TRK-950 (blue) versus control IgG (gray). **C,** ADCC activity against cancer cells. The cytotoxicity of TRK-950 (red) or IgG (black; 0.001–10 μg/mL) in the presence of human NK cells as effector cells was evaluated. Effector/target (E/T) ratio was 25. **D,** ADCC activity against normal cells using the same conditions as described for C. **E,** ADCP activity against cancer cells. The phagocytosis activity of TRK-950 (red) or IgG (black; 0.004–2.5 μg/mL) in the presence of human macrophages as effector cells was evaluated. E/T ratio was 10. Left: Example of phagocytosis analysis by flow cytometry. Target: BT-474, antibody: 2.5 μg/mL TRK-950 or control IgG, effector cells: human macrophages. **F,***In vivo* treatment response. Left: Tumor growth curves of BT-474–bearing NOD-SCID mice with intravenous injection of PBS (vehicle) or TRK-950 treatments at indicated dose once a week for 5 consecutive weeks, Middle: treatment twice a week, Right: treatment once every 2 weeks. Data are given as mean ± SD; *n* = 8/group; *, *P* < 0.05; **, *P* < 0.01; ***, *P* < 0.001; Kruskal–Wallis tests.

On the basis of the rabbit mAb (mAb-1) that reliably identifies CAPRIN-1 on the surface of cancer cell membranes, the humanized anti-CAPRIN-1 antibody (TRK-950) was generated. TRK-950 maintained the specificity of the original antibody and consistently binds to both CAPRIN-1 protein and CAPRIN-1 on the surface of cancer cell membranes (Fig. 6A and B). The binding affinity of TRK-950 to recombinant CAPRIN-1 protein was evaluated using Biacore3000 (Supplementary Fig. S7A), which demonstrated an affinity (*K*_D_) of 3.98 × 10^−13^ (mol/L).

### Mechanism of Action of TRK-950

Because TRK-950 did not directly affect cancer cell growth (Supplementary Fig. S7B), we evaluated the indirect antitumor effects of TRK-950 via immune cells including human NK cells (for ADCC assay) and human monocyte-derived macrophages (for ADCP assay). Importantly, TRK-950 exhibited cytotoxicity and phagocytosis to CAPRIN-1^+^ cancer cells in a dose-dependent fashion (Fig. 6C and E). Notably, TRK-950 did not exhibit cytotoxicity using normal cells (Fig. 6D). We conducted these experiments using NK cells and monocyte-derived macrophages from several donors and obtained similar results.

### Antitumor Effect and Cancer-specific Accumulation of TRK-950 Using *In Vivo* Murine Models

To evaluate the antitumor effect of TRK-950, TRK-950 was administered into BT474–bearing NOD-SCID mice at 100, 30, 10, 5, 3, 1, or 0.3 mg/kg dose once a week. We observed statistically significant inhibition of tumor growth at day 49 compared with the control group. Tumor growth inhibition was also observed in several dose groups in twice a week and every other week administration studies (Fig. 6F). In addition, TRK-950 administration showed tumor growth inhibition in COLO 205 (colon cancer) bearing mouse model (Supplementary Fig. S8A). During these studies, no significant adverse reactions in mice were observed for the TRK-950 groups.

We studied ADCC and ADCP activities using murine effector cells from NOD-SCID mice. We did not detect any ADCC activity of TRK-950 mediated through mouse NK cells (Supplementary Fig. S8B). Conversely, we noticed strong ADCP activity via murine J774.1 cells, mouse BM-derived macrophages (Supplementary Fig. S8C and S8D). Macrophages were the most abundant tumor-infiltrating CD45^+^ cells in the BT-474–bearing mice (Supplementary Fig. S8E). In addition, TRK-950 showed specific ADCP activity via macrophages separated from tumor mass of the mice (Supplementary Fig. S8F). From these data, the action mechanism of the TRK-950 antitumor effect in the xenograft model is considered as an ADCP activity via macrophages.

To confirm the cancer specificity of TRK-950 *in vivo*, IR800Dye-conjugated TRK-950 was administered to BALB/c mice transplanted with CAPRIN-1–expressing mouse 4T1 cells and the *in vivo* localization of the conjugate via imaging was analyzed. One day after administration, the conjugate accumulated in the tumor and remained visible until day 6, suggesting that TRK-950 is recruited to the tumor *in vivo* and shows tumor-specific accumulation (Supplementary Fig. S8G).

### Toxicity Study of TRK-950 in Monkeys

The toxicity of TRK-950 was evaluated using cynomolgus monkeys. CAPRIN-1 is a protein with extremely high interspecies homology, with at least 95% homology (of the amino acid sequence) between humans and mice, and as shown in the above results (Supplementary Fig. S8G), the anti-human CAPRIN-1 antibody (TRK-950) binds to the cell membrane surface of mouse cancer cells (4T1), and therefore it also has binding affinity with mouse CAPRIN-1. CAPRIN-1 of cynomolgus monkeys and that of human share amino acid sequence with a homology of 100% in the main variant and TRK-950 binds to both human and monkey CAPRIN-1, and no concern about cross-reactivity to the target was observed (Supplementary Fig. S9A). In addition, ADCC and ADCP activities of TRK-950 to cancer cells were comparable with those of human and monkey effector cells (Supplementary Fig. S9B and S9C). These results indicated that cynomolgus monkeys are relevant animal for toxicity studies on TRK-950. The maximum dose was set to the highest dose that can be prepared (200 mg/kg), and 3, 25, and 200 mg/kg of TRK-950 (10 mL/kg) were administered on days 1, 8, and 15. Blood was collected when necessary at timepoints specified in the Materials and Method section to evaluate pharmacokinetics/ADA/inflammatory cytokine and blood marker. Pathologic autopsies were performed on days 22 and 72 after the recovery period. Supplementary Tables S1 and S2 show the results of the toxicity study. All animals survived through the test period and tolerated all administered TRK-950 doses. Changes associated with TRK-950 administration were a slight and transient increase in some cytokines (TNFα and IFNγ), and a transient decrease in platelet counts for the maximum dose of 200 mg/kg. A slight increase in the weight of spleen and decrease in the weight of the thyroid and parathyroid were observed, but the dose–response trend was not observed in these changes. The weight of these organs returned to baseline following the 8-week recovery period. In the pharmacokinetic analysis, a minor ADA formation that did not have a significant impact on exposure was observed. At any administered dose, TRK-950 was sufficiently exposed. The exposure of the maximum dose of 200 mg/kg was 396,000 ± 48,000 μgh/mL in male (AUC, 0–168 hours) and 351,000 ± 39,000 μgh/mL in female (AUC, 0–168 hours). The half-life was 4.6 to 7.9 days at 200 mg/kg on day 15. Throughout the test period, TRK-950 was sufficiently exposed, and no significant difference was observed between sexes. When TRK-950 was administered in the dose range as discussed above (3–200 mg/kg), only minor changes were observed and all these changes were reversible, indicating the high tolerability and safety of TRK-950.

## Discussion

Here we demonstrate for the first time that CAPRIN-1 is strongly expressed on the cell membrane surface in most solid cancers, but not normal tissues. When the mRNA expression of CAPRIN-1 was compared between cancer tissues (The Cancer Genome Atlas data) and normal tissues (GTex data) at the GEPIA site, the expression tended to be high in cancer tissues. On the other hand, tissue staining data from Protein Atlas showed the expression of CAPRIN-1 in both normal tissues and cancer tissues with a high score of almost 100%. These do not distinguish between CAPRIN-1 within cells and CAPRIN-1 on the cell membrane. On the other hand, in our data (Fig. 3), mAb1 recognizing CAPRIN-1 on the cell membrane was used and the evaluation was performed under the condition of detecting the expression of CAPRIN-1 on the cell membrane, and a tendency different from the above was observed. CAPRIN-1 was originally identified as a cell membrane protein with a GPI-anchored domain (34). However, it was later determined that the originally published nucleotide sequence was partially wrong, and it was concluded that CAPRIN-1 is a strictly intracellular protein (6). Therefore, it came as a great surprise when we detected CAPRIN-1 on the cell surface and later could indeed validate CAPRIN-1 as a cancer-specific cell surface antigen. The specificity of cancer cell membrane binding anti-CAPRIN-1 antibodies we generated was confirmed by (i) competition assays using soluble recombinant CAPRIN-1 and (ii) abolished binding to CAPRIN-1–deficient cancer cells following *CAPRIN-1* knockdown. As shown in Fig. 2B, CAPRIN-1 proteins in both the cell membrane and cytoplasm were suppressed in *CAPRIN-1* knockdown cells. As a result of the knockdown of *CAPRIN-1* in cancer cells, dead cells increased, suggesting the possibility that CAPRIN-1 is indispensable for cancer cell survival. In flow cytometry analysis, CAPRIN-1 expression on the cell membrane surface was decreased but not abrogated completely in *CAPRIN-1* knocked down cells. We think that the cell membrane surface has a longer turnover time than and may take longer to inhibit expression than CAPRIN-1 in the cytoplasm. Details regarding this can be explained by elucidating the mechanism of membrane expression of CAPRIN-1 in the future.

We confirmed that CAPRIN-1 is present in the cytoplasm, which is consistent with previous reports, and simultaneously determined that a part of the total CAPRIN-1 is actually exposed on the cell membrane surface, a phenomenon that occurs exclusively in cancer cells. Interestingly, analyzing the amino acid sequence using the TMHMM server v.2.0 and SOSUI system we could not predict the formation of transmembrane helices. While these results seem counterintuitive, it is important to note that surface expression of intracellular molecules on cancer cells has been reported previously for various proteins, including heat shock chaperones, cytokeratin, Vimentin, etc. (35). Mechanistically, cell surface exposure can be caused by (i) differential splicing, or (ii) posttranscriptional modifications such as cancer specific glycosylation allowing protein access to the cancer cell surface. Because we did not find differential splicing for CAPRIN-1, we reason that CAPRIN-1 is best classified as a moonlighting protein, which refers to a growing group of intracellular proteins with various nongenetic functions inside and outside the cell (36).

Cancer cells with enhanced stemness features, also referred to as cancer stem cells, are defined by their ability to self-renew and give rise to more differentiated progenies. Moreover, cancer stem cells are highly metastatic, more resistant to anticancer drugs and radiation, thereby driving relapse of disease, and are correlated with poor prognosis (37, 38). While targeting cancer stem cells is a promising therapeutic strategy, current treatments mostly spare this highly aggressive subset of cancer cells, resulting in their relative enrichment and subsequently more aggressive disease during relapse (39). Therefore, we were particularly intrigued to find that CD133^+^ cancer stem cells derived from patients with pancreatic cancer also strongly express CAPRIN-1 on the cell surface. We further corroborated high level expression of CAPRIN-1 on pancreatic cancer stem cells using autofluorescence as a distinct method for detecting cancer stem cells with high accuracy in primary cancer cells (31). Notably, cancer cell surface expression of CAPRIN-1 was not affected by the EMT process, which is an important initial step of the metastatic process. While EMT induction resulted in a marked downregulation of well-known epithelial cancer markers such as E-Cadherin, rendering such epithelial surface markers rather inappropriate for targeting of metastatic disease, there was still high surface expression of CAPRIN-1. Future study is needed to clarify the relationship between stemness or EMT status and CAPRIN-1 membrane surface expression.

In addition, CAPRIN-1^high^ cancer cells have higher *in vitro* colony-forming activity and *in vivo* tumorigenicity compared with CAPRIN-1^low^ cancer cells. The sorted CAPRIN-1^low^ Malme-3M cells tended to have a higher percentage of floating cells than the CAPRIN-1^high^ cells (Supplementary Fig. S5A). This result suggests that the CAPRIN-1 on the cell membrane surface may function in cell adhesion, and this function may be involved in the tumorigenic potential. A hypoxic state is one of the common features of the intratumoral environment (33). Thus, to investigate whether hypoxic conditions affect the expression levels of CAPRIN-1 on the membrane surface of cells, cell lines with high and low expression fractions of CAPRIN-1 on the membrane surface of cells were cultured under hypoxic conditions. The results showed that the proportion of CAPRIN-1^high^ cells increased and the proportion of CAPRIN-1^low^ cells decreased in hypoxic cultures compared with normal cultures. These results suggest that tumor-specific environmental influences, such as hypoxia and other stress stimuli, may cause cancer cells to adapt to the environment and thereby cause CAPRIN-1 to be expressed on the membrane of cells, thereby contributing to malignant transformation to cancer including cancerous proliferation (enhanced tumorigenicity).

On the basis of the capacity of CAPRIN-1 to relocate within the cytoplasm in response to stressful stimuli (12), we hypothesized a potential dynamic translocation of CAPRIN-1 to the cell membrane surface. To explore this concept, we tested various stress stimuli, and found that CAPRIN-1 surface expression was indeed slightly increased in response to ER stress.

Further investigations to gain insights into the mechanisms of how CAPRIN-1 is recruited to the cancer cell membrane and its potential molecular interactions with other surface proteins are warranted and will be the subject of future basic research. Despite these open questions, we felt confident that CAPRIN-1 is a promising target for antibody drugs in solid cancers, and also represents an exciting opportunity for ADCs and CAR-T cells. Therefore, we prepared a humanized anti-CAPRIN-1 mAb namely TRK-950, advanced to the preclinical stage for the treatment of solid cancers.

Preclinical testing was conducted using mouse xenograft models transplanted with human cancer cells, which demonstrated a strong antitumor effect. As expected, in this model ADCC activity via NK cells was greatly reduced because of the functional deficiency of NK cells in NOD-SCID mice. To investigate the antitumor mechanism in this model, we analyzed tumor-infiltrating immune cells and revealed that the main cells were macrophages which showed strong ADCP activity with TRK-950. As it was revealed that TRK-950 showed strong ADCC and ADCP activities against cancer cells via human immune cells *in vitro*, we expect that TRK-950 exerts a high therapeutic effect on patients with cancer by synergistic action of both activities.

Prior to clinical testing we had to ensure safety for use in humans. No overt toxicity up to the highest dose was recorded, and a no-observed-adverse-effect-level (NOAEL) of ≥200 mg/kg/dose was determined. In contrast, previous toxicity studies in non–tumor-bearing monkeys for antibody drugs targeting cancer cell surface antigens such as HER2 (trastuzumab, Herceptin; NOAEL: 59 mg/kg/dose) and CD20 (rituximab, Rituxan; NOAEL ≥20 mg/kg/dose) had indeed shown toxicity (interview form of Herceptin, Chugai Pharmaceutical Co. Ltd.; interview form of Rituxan, Chugai Pharmaceutical Co., Ltd.). Most likely, the toxicity observed in these nonclinical studies was due to shared cancer antigen expression in normal tissues. Therefore, these data indicate that TRK-950 for targeting cell surface CAPRIN-1 provides an excellent safety profile with very low toxicity in cynomolgus monkeys.

Collectively, these data showed that TRK-950 represents a novel and promising cancer drug for clinical applications, a notion that is further supported by the fact that we have already successfully completed a phase I clinical study, and are currently conducting a phase Ib clinical study on TRK-950 as a new cancer drug candidate in combination with conventional cancer drugs.

## Supplementary Material

Figure S1 Anti-CAPRIN-1 antibodies

Figure S2 CAPRIN-1 correlated to the cell proliferation and viability.

Figure S3 CAPRIN-1 is not expressed on the cell surface of hematologic cancer cells.

Figure S4 CAPRIN-1 expression following EMT induction.

Figure S5 Correlation of CAPRIN-1 expression levels on the cell surface with tumor cell growth in vivo or in vitro.

Figure S6 CAPRIN-1 expression on the cell membrane increases due to stressful stimuli to the cancer cells.

Figure S7 Properties of TRK-950

Figure S8 Mechanism of the TRK-950 antitumor effect in mouse model

Figure S9 ADCC and ADCP activity of TRK-950 via human and monkey immune cells

Table S1 Clinical, pathological findings in the toxicity study in Cynomolgus Monkeys

Table S2 Toxicokinetics of TRK-950 in Cynomolgus Monkeys
