# Supplementary material for: Identification of Membrane-expressed CAPRIN-1 as a Novel and Universal Cancer Target, and Generation of a Therapeutic Anti-CAPRIN-1 Antibody TRK-950
Source: Cancer Res Commun. 2023 Apr 18;3(4):640–58. doi: 10.1158/2767-9764.CRC-22-0310 (PMC10112292; doi:10.1158/2767-9764.CRC-22-0310)
Supplement: Figure S1 — Anti-CAPRIN-1 antibodies [file crc-22-0310-s01.pdf]

Fig. S1

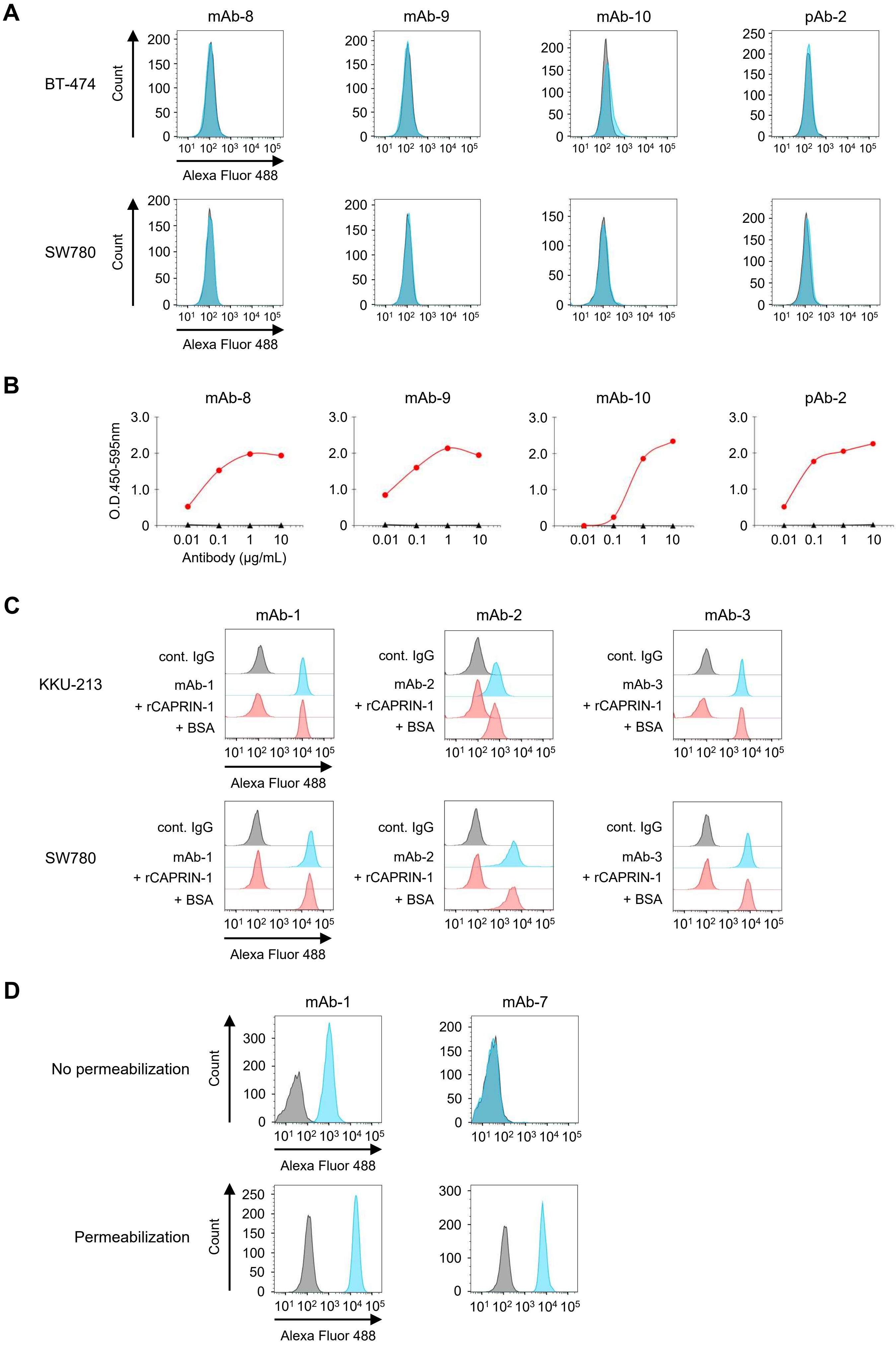
A
mAb-8
mAb-9
mAb-10
pAb-2
BT-474
SW780
Count
Alexa Fluor 488
B
mAb-8
mAb-9
mAb-10
pAb-2
O.D.450-595nm
Antibody (μg/mL)
C
mAb-1
mAb-2
mAb-3
KKU-213
SW780
cont. IgG
mAb-1
mAb-2
mAb-3
+ rCAPRIN-1
+ BSA
Alexa Fluor 488
D
mAb-1
mAb-7
No permeabilization
Permeabilization
Count
Alexa Fluor 488

Supplementary Figure S1. Anti-CAPRIN-1 antibodies

(A) Flow cytometry histograms for anti-CAPRIN-1 monoclonal antibodies mAb-8 to mAb-10, polyclonal antibody pAb-2 (blue), and control IgG (gray). Alexa Fluor 488 anti-rabbit IgG, anti-mouse IgG, or anti-human IgG were used as secondary antibodies.

(B) Anti-CAPRIN-1 antibodies (mAb-8, 9, 10, pAb-2) bind to recombinant CAPRIN-1 protein in ELISA assays. Red line: anti-CAPRIN-1 antibodies, black line: control IgG.

(C) Competition assay with rCAPRIN-1 protein. Binding of mAb-1, 2, 3 to the cell surface of KKU-213 and SW780 cells was analyzed by flow cytometry. Flow cytometry plots for anti-CAPRIN-1 antibodies mAb-1, 2, 3 (blue), control IgG (gray), mAb-1, 2, 3 competed with rCAPRIN-1 or BSA (red).

(D) Cytoplasmic CAPRIN-1 detected by flow cytometry following permeabilization. CAPRIN-1 expression in BT-474 cells without (upper) or with (lower) permeabilization was detected using mAb-1(blue), mAb-5 (blue), or rabbit IgG (gray).
