# Supplementary material for: Identification of Membrane-expressed CAPRIN-1 as a Novel and Universal Cancer Target, and Generation of a Therapeutic Anti-CAPRIN-1 Antibody TRK-950
Source: Cancer Res Commun. 2023 Apr 18;3(4):640–58. doi: 10.1158/2767-9764.CRC-22-0310 (PMC10112292; doi:10.1158/2767-9764.CRC-22-0310)
Supplement: Figure S2 — CAPRIN-1 correlated to the cell proliferation and viability. [file crc-22-0310-s02.pdf]

Fig. S2

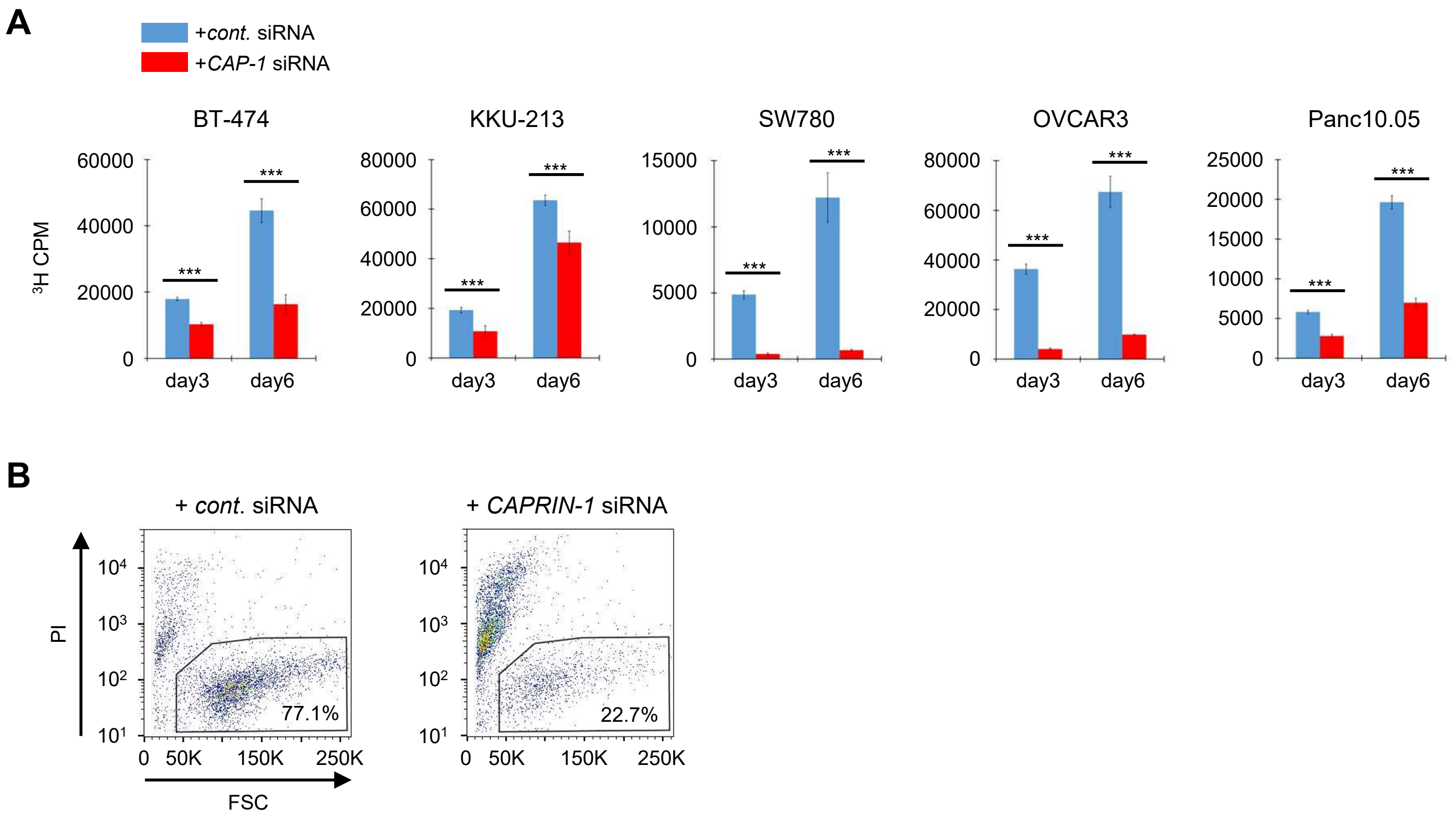

Supplementary Figure S2. CAPRIN-1 correlated to the cell proliferation and viability.

(A) Association of CAPRIN-1 with cell proliferation. Cell proliferation was measured by 3H-labeled thymidine incorporation 3 or 6 days after transfection of control siRNA (blue) or CAPRIN-1 siRNA (red) into five indicated cell lines. Data are displayed as mean $\pm$ SD; n=3; ***p < 0.001, two-tailed student's t-test.

(B) Association of CAPRIN-1 with cell viability. Cell viability 3 days after transfection of control siRNA or CAPRIN-1 siRNA into OVCAR3. The percentage of alive cells was analyzed by flow cytometry using PI dye exclusion.
