# Supplementary material for: Identification of Membrane-expressed CAPRIN-1 as a Novel and Universal Cancer Target, and Generation of a Therapeutic Anti-CAPRIN-1 Antibody TRK-950
Source: Cancer Res Commun. 2023 Apr 18;3(4):640–58. doi: 10.1158/2767-9764.CRC-22-0310 (PMC10112292; doi:10.1158/2767-9764.CRC-22-0310)
Supplement: Figure S3 — CAPRIN-1 is not expressed on the cell surface of hematologic cancer cells. [file crc-22-0310-s03.pdf]

Fig. S3

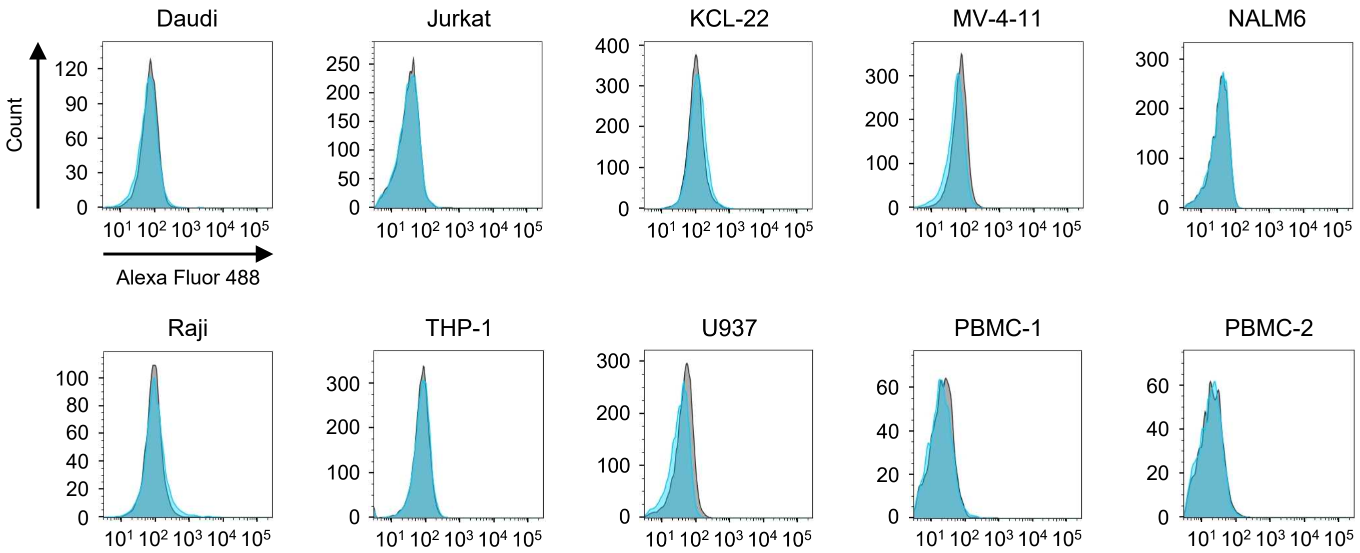

Supplementary Figure S3. CAPRIN-1 is not expressed on the cell surface of hematologic cancer cells.

CAPRIN-1 expression on the cell membrane surface of hematologic cancer cells and human PBMCs was analyzed by flow cytometry using mAb-1 (blue) and rabbit IgG (gray).
