# Supplementary material for: Identification of Membrane-expressed CAPRIN-1 as a Novel and Universal Cancer Target, and Generation of a Therapeutic Anti-CAPRIN-1 Antibody TRK-950
Source: Cancer Res Commun. 2023 Apr 18;3(4):640–58. doi: 10.1158/2767-9764.CRC-22-0310 (PMC10112292; doi:10.1158/2767-9764.CRC-22-0310)
Supplement: Figure S4 — CAPRIN-1 expression following EMT induction. [file crc-22-0310-s04.pdf]

Fig. S4

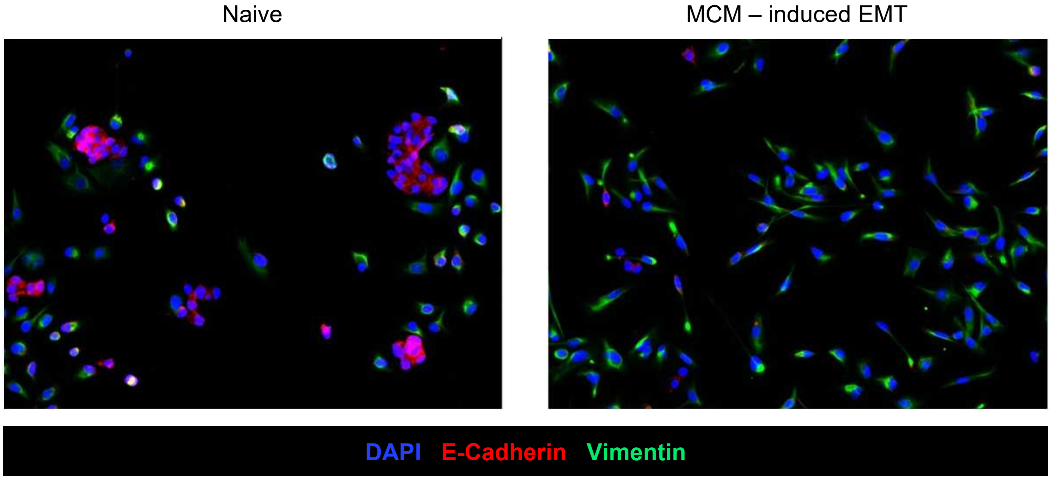

Supplementary Figure S4. CAPRIN-1 expression following EMT induction.
Naive cells (human primary pancreatic cancer cells, SiC-003) were cultured with macrophage conditioned medium to induce EMT. Immunofluorescent staining was conducted before and after EMT induction.
