# Supplementary material for: Identification of Membrane-expressed CAPRIN-1 as a Novel and Universal Cancer Target, and Generation of a Therapeutic Anti-CAPRIN-1 Antibody TRK-950
Source: Cancer Res Commun. 2023 Apr 18;3(4):640–58. doi: 10.1158/2767-9764.CRC-22-0310 (PMC10112292; doi:10.1158/2767-9764.CRC-22-0310)
Supplement: Figure S5 — Correlation of CAPRIN-1 expression levels on the cell surface with tumor cell growth in vivo or in vitro. [file crc-22-0310-s05.pdf]

Fig. S5

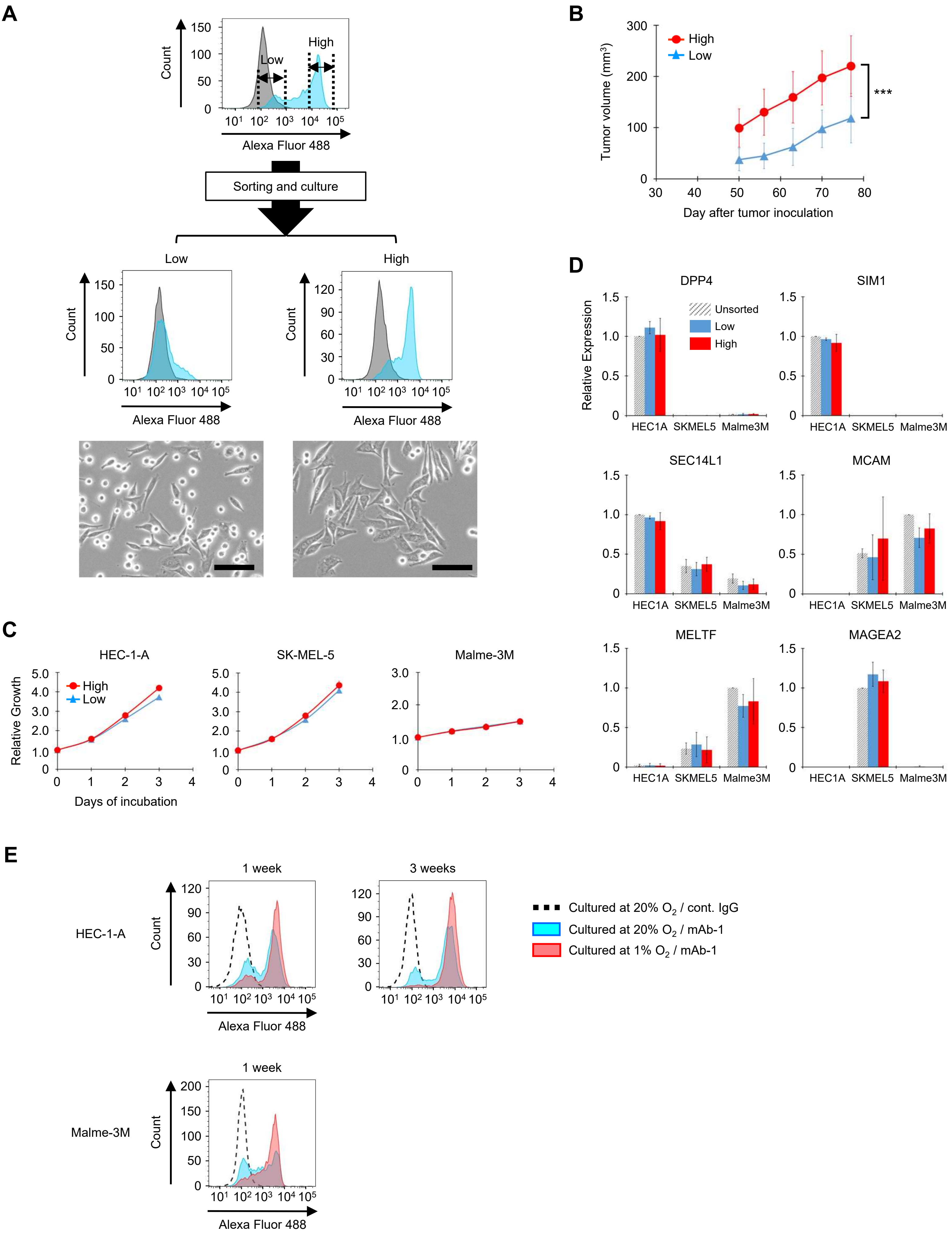

A
Count
150
100
50
0
Low
High
10¹ 10² 10³ 10⁴ 10⁵
Alexa Fluor 488
Sorting and culture
Low
High
Count
120
90
60
30
B
300
200
100
0
High
Low
Tumor volume (mm³)
***
30 40 50 60 70 80
Day after tumor inoculation
C
HEC-1-A
SK-MEL-5
Malme-3M
Relative Growth
5.0
4.0
3.0
2.0
1.0
Days of incubation
D
DPP4
SIM1
Unsorted
Relative Expression
1.5
1.0
0.5
HEC1A SKMEL5 Malme3M
SEC14L1
MCAM
MELTF
MAGEA2
E
1 week
3 weeks
HEC-1-A
Malme-3M
Cultured at 20% O₂ / cont. IgG
Cultured at 20% O₂ / mAb-1
Cultured at 1% O₂ / mAb-1

Supplementary Figure S5. Correlation of CAPRIN-1 expression levels on the cell surface with tumor cell growth in vivo or in vitro.

(A) Flow cytometry analysis of Malme-3M cells before and after sorting using mAb-1 (blue) and rabbit IgG (gray). The micrographs display the sorted cells cultured for 10 days. Scale bar is 100 μm.

(B) Tumor growth curves of NOD-SCID mice bearing CAPRIN-1^{high} (red circles) and CAPRIN-1^{low} (blue triangles) Malme-3M cells. Tumor measurement started on day 50. Data are given as mean ± SD; n=10/group; ***$p < 0.001$, Mann-Whitney U tests.

(C) Anchorage-dependent growth assay (regular plate culture) of sorted HEC-1-A, SK-MEL-5, and Malme-3M cells with high (red line) and low (blue line) expression of CAPRIN-1.

(D) mRNA expression levels of cell-type specific genes in unsorted cells (hatched), and sorted CAPRIN-1^{high} (red) and CAPRIN-1^{low} (blue) analyzed by qPCR. Data are shown as mean ± SD; n=3.

(E) After culturing HEC-1-A and Malme-3M cells under hypoxic condition (1% O_2) for 1-3 weeks, CAPRIN-1 expression on the surface were analyzed by flow cytometry using mAb-1. Blue: cultured under normoxic condition (20% O_2) cells, red: cultured under hypoxic condition cells.
