# Supplementary material for: Identification of Membrane-expressed CAPRIN-1 as a Novel and Universal Cancer Target, and Generation of a Therapeutic Anti-CAPRIN-1 Antibody TRK-950
Source: Cancer Res Commun. 2023 Apr 18;3(4):640–58. doi: 10.1158/2767-9764.CRC-22-0310 (PMC10112292; doi:10.1158/2767-9764.CRC-22-0310)
Supplement: Figure S6 — CAPRIN-1 expression on the cell membrane increases due to stressful stimuli to the cancer cells. [file crc-22-0310-s06.pdf]

Fig. S6

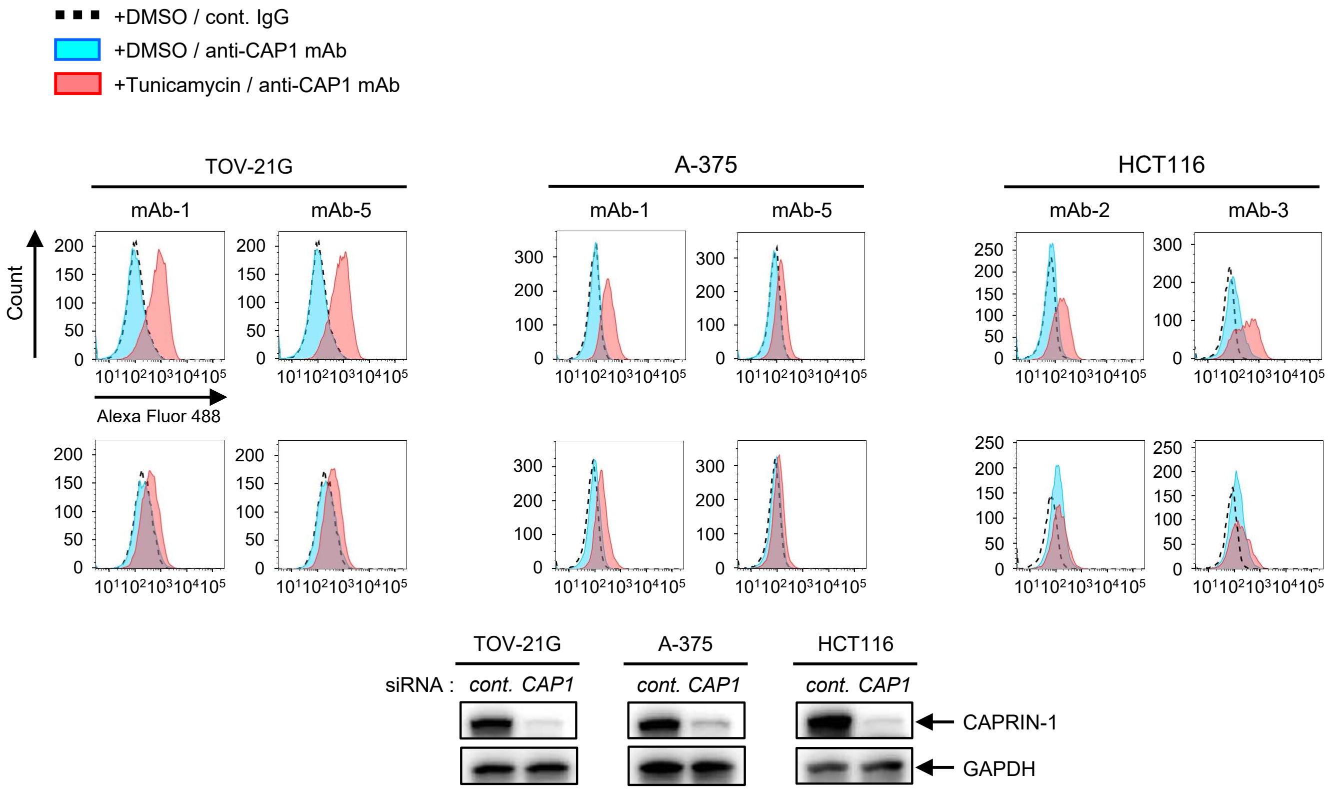

Supplementary Figure S6. CAPRIN-1 expression on the cell membrane increases due to stressful stimuli to the cancer cells.

Induction of CAPRIN-1 surface expression by stress stimuli. TOV-21G, A-375, and HCT116 cells were treated with 1.5 μM Tunicamycin or DMSO (control) for 24 hours. CAPRIN-1 expression on the cell membrane surface was analyzed by flow cytometry using the indicated anti-CAPRIN-1 antibodies (blue: DMSO treated cells, red: tunicamycin treated cells), and rabbit IgG (dotted line). Comparison of CAPRIN-1 siRNA (lower) and control siRNA (upper) transfected cells 48 hours after siRNA introduction. Western blot was conducted at 48 hours after siRNA introduction.
