# Supplementary material for: Identification of Membrane-expressed CAPRIN-1 as a Novel and Universal Cancer Target, and Generation of a Therapeutic Anti-CAPRIN-1 Antibody TRK-950
Source: Cancer Res Commun. 2023 Apr 18;3(4):640–58. doi: 10.1158/2767-9764.CRC-22-0310 (PMC10112292; doi:10.1158/2767-9764.CRC-22-0310)
Supplement: Figure S7 — Properties of TRK-950 [file crc-22-0310-s07.pdf]

Fig. S7

A

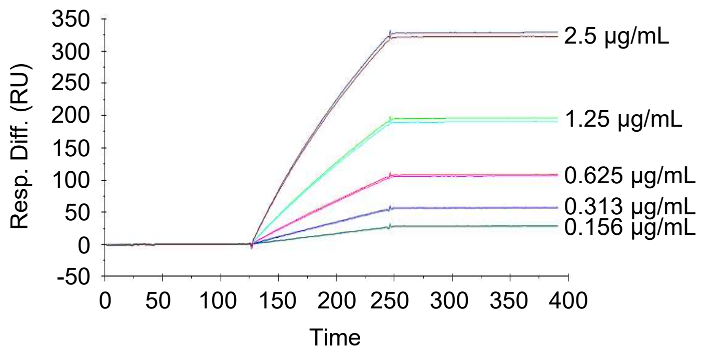

k_a (1/Ms)	k_d (1/s)	K_A (1/M)	K_D (M)
3.91×10^{5}	1.55×10^{-7}	2.52×10^{12}	3.98×10^{-13}

B

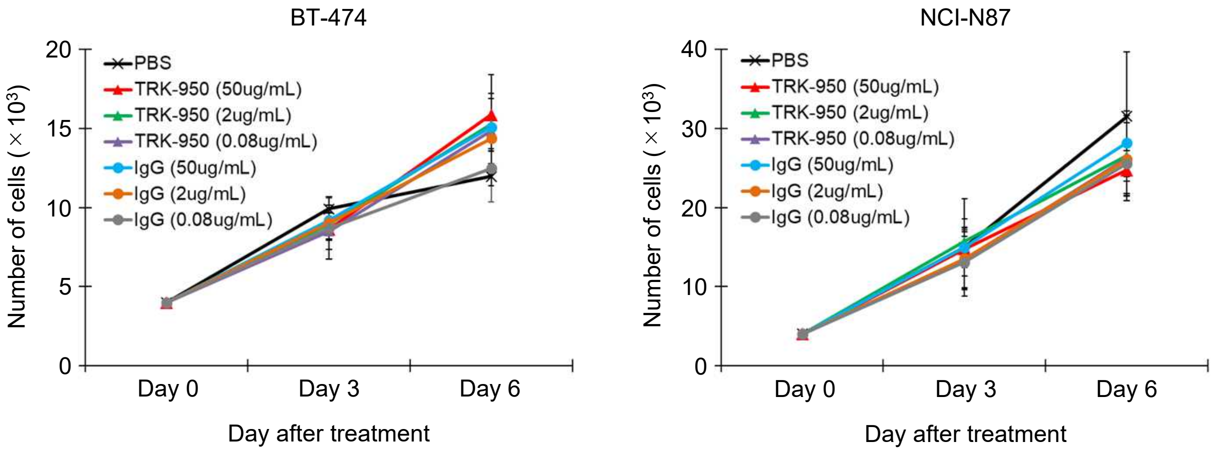

Supplementary Figure S7. Properties of TRK-950

(A) Binding affinity of TRK-950 (0.158 - 2.5 µg/mL) to rCAPRIN-1 protein was analyzed with Biacore. The response was measured in duplicate.

Resp. Diff.: response difference [response to rCAPRIN-1] – [response to BSA]

(B) The direct effect of TRK-950 (0.08, 2, 50 µg/mL) to the cell growth of cancer cells. No significant in two-tailed student's t-test. Data are shown as mean ± SD; n=4.
