# Supplementary material for: Identification of Membrane-expressed CAPRIN-1 as a Novel and Universal Cancer Target, and Generation of a Therapeutic Anti-CAPRIN-1 Antibody TRK-950
Source: Cancer Res Commun. 2023 Apr 18;3(4):640–58. doi: 10.1158/2767-9764.CRC-22-0310 (PMC10112292; doi:10.1158/2767-9764.CRC-22-0310)
Supplement: Figure S8 — Mechanism of the TRK-950 antitumor effect in mouse model [file crc-22-0310-s08.pdf]

Fig. S8

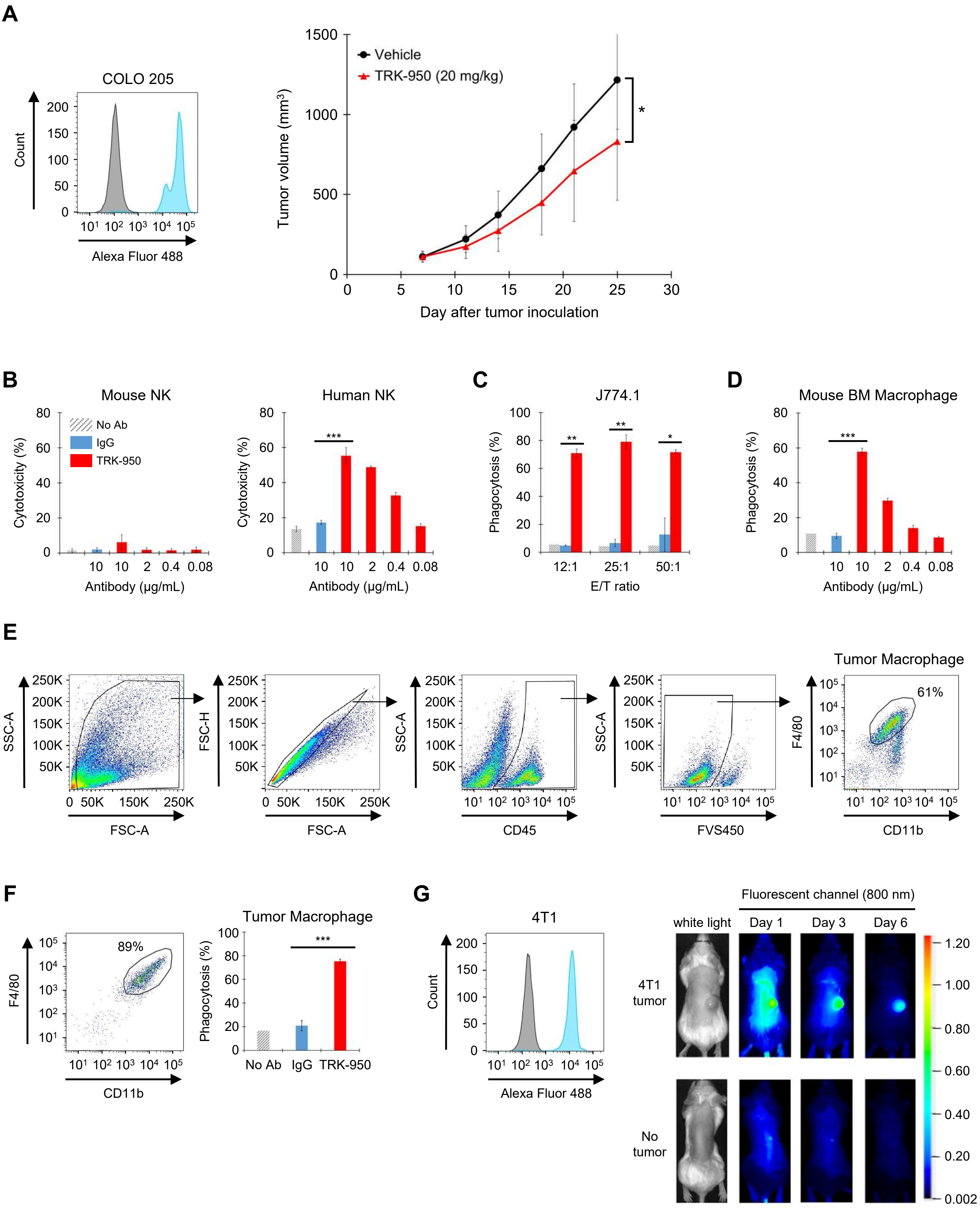
A
COLO 205
Count
Alexa Fluor 488
Vehicle
TRK-950 (20 mg/kg)
Tumor volume (mm³)
Day after tumor inoculation
B
Mouse NK
Human NK
No Ab
IgG
TRK-950
Cytotoxicity (%)
Antibody (μg/mL)
C
J774.1
Phagocytosis (%)
E/T ratio
D
Mouse BM Macrophage
E
Tumor Macrophage
61%
SSC-A
FSC-A
FSC-H
CD45
FVS450
F4/80
CD11b
F
89%
Tumor Macrophage
No Ab
IgG
TRK-950
G
4T1
Fluorescent channel (800 nm)
white light
Day 1
Day 3
Day 6
4T1 tumor
No tumor

Supplementary Figure S8. Mechanism of the TRK-950 antitumor effect in mouse model.

(A) Left: CAPRIN-1 on the membrane of COLO 205 cell was analyzed by flow cytometry using TRK-950 (blue) and human IgG (gray). Right: In vivo treatment response. Tumor growth curves of COLO 205 bearing NOD-SCID mice with IV injection of PBS (vehicle) or TRK-950 treatments at indicated dose once a week for 3 consecutive weeks. Treatment stated at day 0. Data are given as mean $\pm$ SD; n=7/group; *$p < 0.05$, Mann-Whitney U tests.

(B) ADCC activity against BT-474 cells via NOD-SCID mouse NK or human NK cells in the presence of indicated concentrations of TRK-950 (red), control IgG (blue), or no antibody (hatched). E/T ratio was 25. Data are displayed as mean $\pm$ SD; n=3.

(C and D) B: ADCP activity of 10 μg/mL TRK-950 (red), control IgG (blue), or no antibody (hatched) against BT-474 cells via J774.1 cells, C: via mouse bone marrow-derived (BM) macrophage from NOD-SCID, E/T ratio was 25. Data are displayed as mean $\pm$ SD; n=3.

(E) Tumor infiltrating cells from BT-474 bearing NOD-SCID mice were analyzed by flow cytometry using the shown gating strategy. Macrophage was defined by $CD11b^+$, $F4/80^{high}$ in singlet cells, $CD45^+$, alive cell population.

(F) Left: The purity of isolated tumor infiltrating macrophages (tumor macrophages) from BT-474 bearing NOD-SCID mice was analyzed using the same method as (D). Right: ADCP activity against BT-474 cells via the tumor macrophages in the presence of 10 μg/mL TRK-950 (red), control IgG (blue), or no antibody (hatched). E/T ratio was 25. Data are displayed as mean $\pm$ SD; n=3.

(G) Left: CAPRIN-1 on the membrane of murine 4T1 cell was analyzed by flow cytometry using TRK-950 (blue) and human IgG (gray). Right: Tumor localization of intravenously administered TRK-950-IRDye800 in 4T1 bearing BALB/c mice at 1, 3, 6 days after antibody injection.

Significance calculated using two-tailed student's t-test (B, C, D, F); *$p < 0.05$, **$p < 0.01$, ***$p < 0.001$.
