# Supplementary material for: Identification of Membrane-expressed CAPRIN-1 as a Novel and Universal Cancer Target, and Generation of a Therapeutic Anti-CAPRIN-1 Antibody TRK-950
Source: Cancer Res Commun. 2023 Apr 18;3(4):640–58. doi: 10.1158/2767-9764.CRC-22-0310 (PMC10112292; doi:10.1158/2767-9764.CRC-22-0310)
Supplement: Figure S9 — ADCC and ADCP activity of TRK-950 via human and monkey immune cells [file crc-22-0310-s09.pdf]

**Fig. S9**

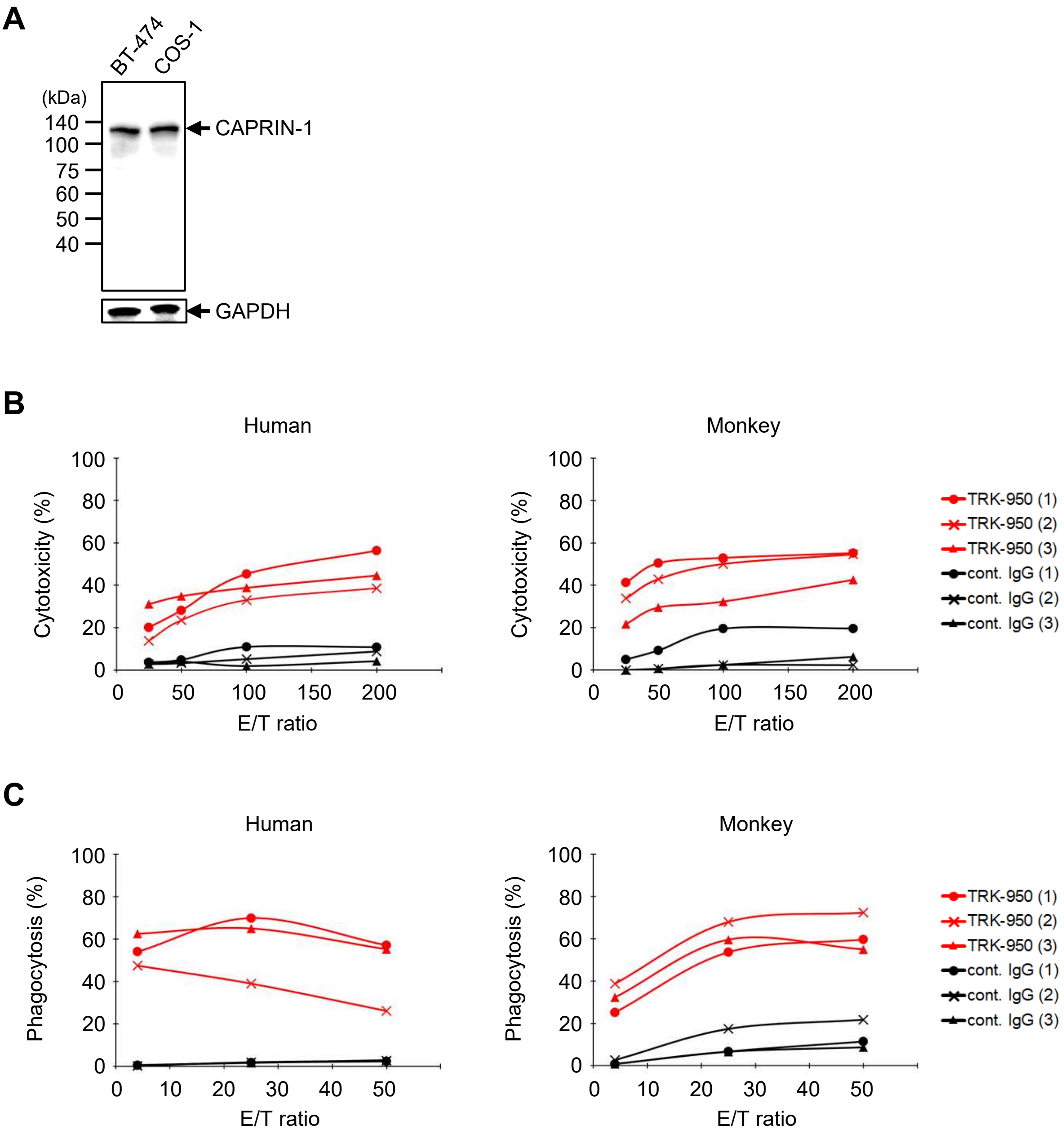


**Supplementary Figure S9. ADCC and ADCP activity of TRK-950 via human and monkey immune cells.**

**(A)** CAPRIN-1 proteins in lysates from human BT-474 cells and monkey COS-1 cells were detected by Western Blot using TRK-950.

**(B)** ADCC activity of TRK-950 against BT-474 cells via human PBMCs or cynomolgus monkey PBMCs in the presence of 1 μg/mL TRK-950 (red) or human IgG (black) at 4 different E/T ratios (25, 50, 100 and 200). PBMCs were obtained from three individual human and monkey donors.

**(C)** ADCP activity of TRK-950 against BT-474 cells via human neutrophils or cynomolgus monkey neutrophils in the presence of 1 μg/mL TRK-950 (red) or human IgG (black) at 3 different E/T ratios (4, 25, and 50). Neutrophils were obtained from three individual human and monkey donors.
