# Supplementary material for: Identification of Membrane-expressed CAPRIN-1 as a Novel and Universal Cancer Target, and Generation of a Therapeutic Anti-CAPRIN-1 Antibody TRK-950
Source: Cancer Res Commun. 2023 Apr 18;3(4):640–58. doi: 10.1158/2767-9764.CRC-22-0310 (PMC10112292; doi:10.1158/2767-9764.CRC-22-0310)
Supplement: Table S1 — Clinical, pathological findings in the toxicity study in Cynomolgus Monkeys [file crc-22-0310-s10.pdf]

Supplementary Table S1. Clinical, pathological findings in the toxicity study in Cynomolgus Monkeys.

Dose (mg/kg/dose)	0		3		25		200	
	Male	Female	Male	Female	Male	Female	Male	Female
Clinical observations	AC	AC	AC	AC	AC	AC	AC	AC
Ophthalmoscopy	AC	AC	AC	AC	AC	AC	AC	AC
Electrocardiography	AC	AC	AC	AC	AC	AC	AC	AC
Hematology	AC	AC	AC	AC	AC	AC	P- *1*5	P- *2*5
Serum chemistry	AC	AC	AC	AC	AC	AC	AC	AC
Cytokine release	AC	AC	AC	AC	AC	AC	T+ *3*5<br>I+ *4*5	T+ *3*5<br>I+ *4*5
Urinalysis	AC	AC	AC	AC	AC	AC	AC	AC
Organ weights	AC	AC	S+	TA-	S+<br>TA-	TA-	S+ *5<br>TA- *5	TA- *5
Gross pathology	AC	AC	AC	AC	AC	AC	AC	AC
Histopathology	AC	AC	AC	AC	AC	AC	AC	AC
Postdose evaluation	AC	AC	AC	AC	AC	AC	AC	AC
Anti-drug antibodies	0/5	0/5	0/3	0/3	0/3	0/3	0/5	2/5 *6
Immunophenotyping	AC	AC	AC	AC	AC	AC	AC	AC

The toxicity of TRK-950 was evaluated using cynomolgus monkeys. All animals survived through the test period and tolerated all administered TRK-950 doses (3 to 200 mg/kg).
AC; absent of change, Abbreviation & +/-; P=platelets, T= TNF-alpha, I=IFN-gamma, S=spleen, TA=Thyroid & parathyroid, +; transient increase, -; transient decrease
Values represent the number of animals with findings. *1; Day 1-15 [0.69 × control animals], *2; Day 8, Day15 [0.49 × control animals], *3; Day1, Day 15 [1h post dose to 24h], Day15 [1h post dose to 24h], <385 pg/mL, *4; marginal increase at from 8 to day 15, <58.9 pg/mL, *5; Full recovery was demonstrated following the 8 weeks cessation of treatment., *6; Day43, Day71.
