# Supplementary material for: Identification of Membrane-expressed CAPRIN-1 as a Novel and Universal Cancer Target, and Generation of a Therapeutic Anti-CAPRIN-1 Antibody TRK-950
Source: Cancer Res Commun. 2023 Apr 18;3(4):640–58. doi: 10.1158/2767-9764.CRC-22-0310 (PMC10112292; doi:10.1158/2767-9764.CRC-22-0310)
Supplement: Table S2 — Toxicokinetics of TRK-950 in Cynomolgus Monkeys [file crc-22-0310-s11.pdf]

Supplementary Table S2. Toxicokinetics of TRK-950 in Cynomolgus Monkeys

Dose (mg/kg/dose)	3		25		200	
	Male	Female	Male	Female	Male	Female
Day 1 C_{max} (ng/mL)	65700	73700	612000	619000	3320000	3160000
Day 15 C_{max} (ng/mL)	104000	97400	786000	772000	4650000	3680000
Day 1 C_{168} (ng/mL)	21500	20400	187000	177000	1230000	941000
Day 15 C_{168} (ng/mL)	24600	30100	203000	257000	1460000	140000
Day 1 AUC_{0-168}(μg·hr/mL)	5790	5630	53300	49100	303000	237000
Day 15 AUC_{0-168} (μg·hr/mL)	8100	8410	61900	70900	396000	351000

Toxicokinetics of three-weeks-toxicology study in cynomolgus monkeys.
Exposure to TRK-950 was confirmed in all animals that received the treatment (three animals for each dose/sex). Maximum serum concentrations (C_{max}) of TRK-950 and the areas under the serum concentration-time curves estimated up to 168 hours post dose (AUC_{0-168}) on Day 1 and Day 15 are described as mean value. The samples which were detected/suspected of the ADA rising are excluded from the determination of the values.
